# Perfluorocarbons: A perspective of theranostic applications and challenges

**DOI:** 10.3389/fbioe.2023.1115254

**Published:** 2023-08-03

**Authors:** Nasrin Kakaei, Roshanak Amirian, Mehdi Azadi, Ghobad Mohammadi, Zhila Izadi

**Affiliations:** ^1^ Student Research Committee, School of Pharmacy, Kermanshah University of Medical Sciences, Kermanshah, Iran; ^2^ USERN Office, Kermanshah University of Medical Sciences, Kermanshah, Iran; ^3^ Pharmaceutical Sciences Research Center, Health Institute, Kermanshah University of Medical Sciences, Kermanshah, Iran

**Keywords:** perfluorocarbon (PFC), oxygen carrier, theranostic, imaging, nanoparticles

## Abstract

Perfluorocarbon (PFC) are biocompatible compounds, chemically and biologically inert, and lacks toxicity as oxygen carriers. PFCs nanoemulsions and nanoparticles (NPs) are highly used in diagnostic imaging and enable novel imaging technology in clinical imaging modalities to notice and image pathological and physiological alterations. Therapeutics with PFCs such as the innovative approach to preventing thrombus formation, PFC nanodroplets utilized in ultrasonic medication delivery in arthritis, or PFC-based NPs such as Perfluortributylamine (PFTBA), Pentafluorophenyl (PFP), Perfluorohexan (PFH), Perfluorooctyl bromide (PFOB), and others, recently become renowned for oxygenating tumors and enhancing the effects of anticancer treatments as oxygen carriers for tumor hypoxia. In this review, we will discuss the recent advancements that have been made in PFC’s applications in theranostic (therapeutics and diagnostics) as well as assess the benefits and drawbacks of these applications.

## Graphical Abstract

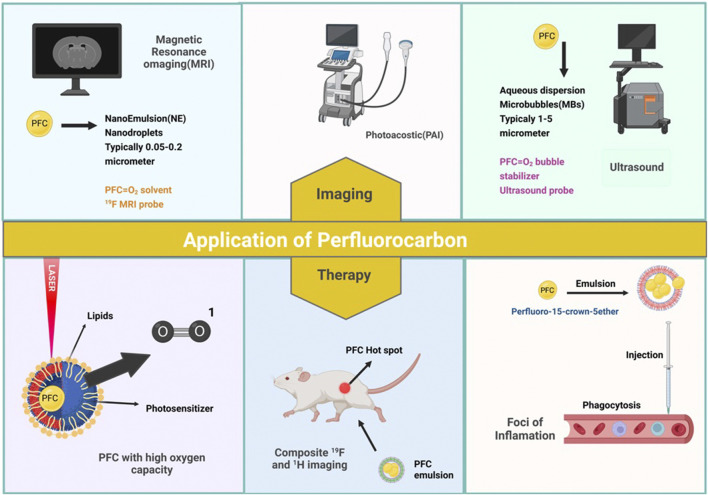

## Highlights

• Perfluorocarbon (PFC) are biocompatible compounds, chemically and biologically inert, and lacks toxicity as oxygen carriers.
• PFC’s applications is in theranostics (therapeutics and diagnostics).
• Numerous diagnostic and therapeutic applications exist for PFCs, including oxygenation, cancer treatment, cell therapy, and imaging.
• PFCs NPs are employed in ultrasound and MRI to label cells, target distinct epitopes in the tumor, monitor treatment effectiveness, quantify tumor characteristics, and detect changes in the tumor's surrounding environment.
• PFC-based NPs become renowned for oxygenating tumors and enhancing the effects of anticancer treatments as oxygen carriers for tumor hypoxia.
• Clinical translation of imaging technologies using PFC should be carefully examined, and long-term toxicity problems should be investigated.

## 1 Introduction

Perfluorocarbons (PFCs) are odorless, non-corrosive, colorless liquids with low surface tension and a considerable density difference with air. Their density is almost double that of water, and they are highly stable and miscible with biological fluids. In terms of chemical composition, they are hydrocarbons in which fluorine replaces most or all of the hydrogen atoms, and, occasionally, other halogen atoms are present in their structure (Xiang et al., 2019; Charbe et al., 2022). These substitutions change the physical properties of these compounds. The element with the highest electronegativity is the fluorine (Ahrens and Zhong, 2013; Li et al., 2022). As a result, the carbon-fluorine bond in these compounds is powerful and polar, but it does not result in water solubility because the molecule is ultimately non-polar. All PFC molecules can dissolve vast quantities of gas. Table 1 compares the oxygen dissolution rates of two of the most commonly used PFCs, perfluorooctyl bromide (PFB) and perfluorodecalin (PFD). Besides oxygen, these compounds can dissolve up to four times as much CO_2_ as oxygen. A liter of water contains 55 mol, whereas a liter of PFD only contains 4.2 mol. Therefore, the molecular ratio of O_2_ dissolved in 1_O2_: Is 200 water in water, but PFD equals 5_O2_: 1_PFD_. This demonstrates that the PDF molecule is 1,000 times more soluble than water (Charbe et al., 2022; Mohanto et al., 2023).

**TABLE 1:** Comparison of the main physical properties of water and PFCs.

	Water	Perfluorooctyl bromide (PFOB)	Perfluorodecalin (PFD)	Perfluorotributylamine (PFTBA)
Formula	H_2_O	C_8_BrF_17_	C_10_F_18_	C_12_F_27_N
Molar mass	18 g/mol	499 g/mol	462 g/mol	671 g/mol
Density	0.997 g/cm^3^	1.89 g/cm^3^	1.946 g/cm^3^	1.884 g/cm^3^
Molar density	55.4 mol/L	3.8 mol/L	4.2 mol/L	2.8 mol/L
Oxygen solubility (25°C)	6.3 mLO_2_/LH_2_O	527 mLO_2_/LPFOB	403 mLO_2_/LPFD	—

PFCs are biocompatible compounds because they are both chemically and biologically inert (Jagers et al., 2020). In addition to lacking toxicity, carcinogenicity, mutagenicity, and teratogenicity. PFCs eliminate from the body by the reticuloendothelial system, the lungs, and, to a lesser extent, the skin (Lambert et al., 2019). Their tissue half-lives range from 4 to 65 days for perfluorooctyl bromide and perfluorotripropylamine, respectively.

The biocompatibility of PFCs has been studied in both *in vitro* and *in vivo* models, and the results have been mixed (Lauby et al., 2022; Mohanto et al., 2023). Wrobeln et al. (2017) *In vivo* evaluation demonstrated the least but dose-dependent side-effects such as the peak of plasma concentration of cellular enzymes.

In widespread, PFCs are well-tolerated when utilized as a blood alternate (Abutarboush et al., 2016), although some studies have reported toxicity or adverse immune reactions *in-vitro* models (Menz et al., 2018). In the duration of oxygen delivery, PFC-based emulsions are effective in improving oxygenation in animals with lung injury or hypoxia (Liu et al., 2022; Luo et al., 2023). Nevertheless, the long-term safety and efficacy of PFC-based oxygen carriers have not been comprehensively confirmed (Alayash, 2014; Charbe et al., 2022).

Prevailing, PFCs show assurance as a potential implement for medical applications, but further research is needed to fully understand their biocompatibility and to specify the most suitable uses for these materials.

PFCs can be used in emulsions, nanoemulsions, and gases. Numerous diagnostic and therapeutic applications exist for PFCs, including oxygenation, cancer treatment, cell therapy, and imaging. In this article, we will evaluate the recent advancements that have been made in PFC’s applications in diagnosis and treatment, as well as will discuss the benefits and drawbacks of these applications.

## 2 Application of PFCs in imaging

**FIGURE 1:**
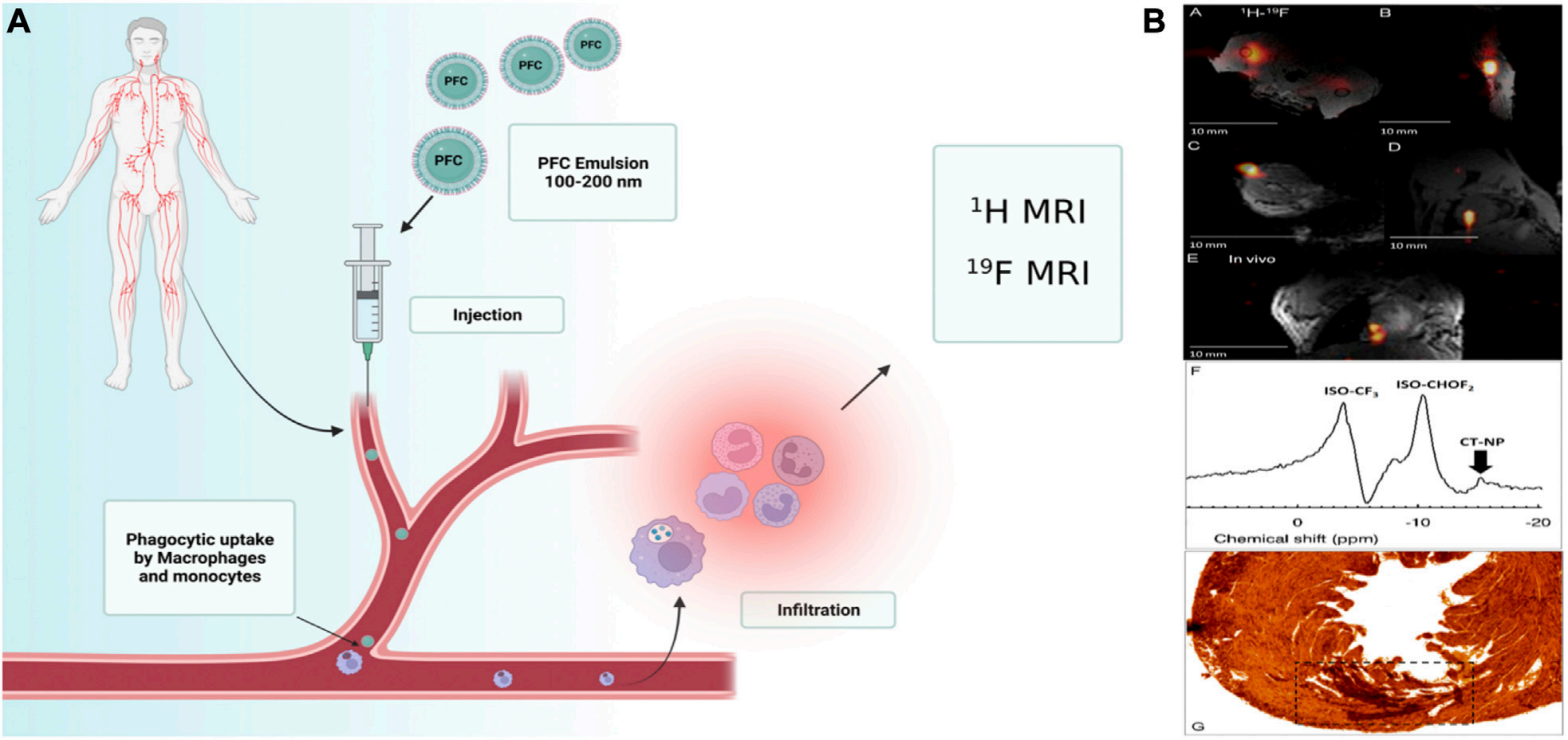
**(A)**Schematic illustration of a multifunctional liquid perfluorocarbon nanoemulsions (100–200 nm) which can be modified with another cargo targeting ligands such as aptamers and polysaccharides, intravenous injection, and then directs cellular uptake by circulating or provincial phagocytic, immune cells such as monocytes and macrophages; MRI detects the collection of these cells. **(B)** Post-mortem and *in vivo* murine cardiac 19F MRI following intramyocardial CPC injections. Reprinted with permission from Ref (Constantinides et al., 2018). Copyright (2018) by the Public Library of Science (PLOS).

For diagnostic imaging, numerous NPs and microparticles are used. PFC NPs are a novel imaging technology used in clinical imaging modalities. Multiple PFC NPs detect and image pathological and physiological alterations. The long-term systematic half-life of PFC NPs, which permits long-term binding to ligands, makes PFCs suitable for imaging. These NPs are employed in ultrasound and MRI techniques (Ahrens and Zhong, 2013). These molecular imaging probes are primarily used in MRI to label cells, target specific epitopes in the tumor, monitor treatment efficacy, quantify tumor features, and detect changes in the tumor’s surrounding environment (Figures 1A) (Barnett et al., 2011; Cosco et al., 2015; Vidallon et al., 2022).

It is crucial to comprehend the NMR phenomenon to understand how PFC NPs are used as a contrast agent in the MR technique. When exposed to a strong magnetic field, the nuclei of elements such as ^1^H, ^13^C, and ^19^F change from random to parallel or antiparallel in nuclear magnetic resonance. The energy level increases as the nucleus absorb the radio frequency waves’ energy, then core returns to a lower energy level following excitement. This distinction is referred to as magnetic resonance intensification (T1). This process of excitation and relaxation depends on the external magnetic field. When nuclei interact, the signal strength decreases, a phenomenon known as the T2 constant. MR imaging can determine the T1 and T2 relaxation times, densities, and exposure intensities. MR contrast agents function by reducing T1 and T2. The most frequently used non-targeted MR contrast agents are paramagnetic ions (gadolinium chelates) which lessen the T1 relaxation time. Compounds that are paramagnetic or super-paramagnetic have a high magnetic sensitivity and cause field disturbances. This disorder causes dephasing of the signal in the tissues and signal loss due to the loss of T2. Unlike T1 contrast materials, super-paramagnetic agents have a net effect on their environment.

NPs of PFCs are superficially functionalized with ligand, precisely one hundred thousand chelates of gadolinium per particle, to obtain T1 when employing paramagnetic contrast agents. Present paramagnetic ions present relaxivity, which is obtained by dividing the change in comfort velocity (1/T1 or 1/T2) by the concentration of the contrast agent, which describes the performance of the MR contrast agent. At 1.5 T, the Gd+3 ions in saline have a lower relaxivity than those bonded to the surface of PFC NPs. Each nanoparticle carries numerous gadolinium ions; the structural relaxivity is proportional to the particle relaxivity and is measured at 2,000,000 mM^−1^s^−1^. It is, therefore, possible to detect and quantify biomarkers at nano concentrations (Tran et al., 2007; Wu et al., 2020).

Various tissues’ T1 and T2 relaxation times vary based on the surrounding water and proton content. Because of its natural frequency, gyromagnetic ratio, and high concentration in biological tissues, proton 1H is one of the most widely employed nuclei in medical imaging. ^19^F has a near-proton gyromagnetic ratio and nearly 100 percent natural abundance, making it an attractive nucleus for MR imaging (Bouchlaka et al., 2016). In a field of equal strength and the number of equivalent nuclei, its sensitivity is 83% compared to proton nuclei. The concentration of ^19^F ions in biological tissues is low; therefore, if the tissue is not enriched with a^19^F contrast agent, the resulting image will be unsuitable. As this contrast factor increases, so does the concentration of ^19^F in the environment of biological tissue. Under these conditions, imaging will be possible without background signal interference. ^19^F has seven outer electrons, whereas hydrogen has only one, so the chemical shift around fluorine is more significant than that around hydrogen. ^19^F nuclei exhibit a wide range of chemical changes (>350 ppm) and are highly sensitive to relaxation changes, resulting in a higher resolution than HMRI. Therefore, if several different types of PFCs are present simultaneously, they can be detected by MR and imaging due to the chemical difference.

PFCs are neither metabolized nor collapsed by lysosomal enzymes. Table.3 summarizes the imaging applications of PFCs. For example, cardiac progenitor stem cells (CPCs) and bone marrow macrophages labeled with perfluoro-crown-ether (PFCE), then performed ^19^F-Magnetic Resonance Imaging (MRI). Limitation of cell load and determination of label concentration were other goals of this study (Figures 1B) (Constantinides et al., 2018).

**FIGURE 2:**
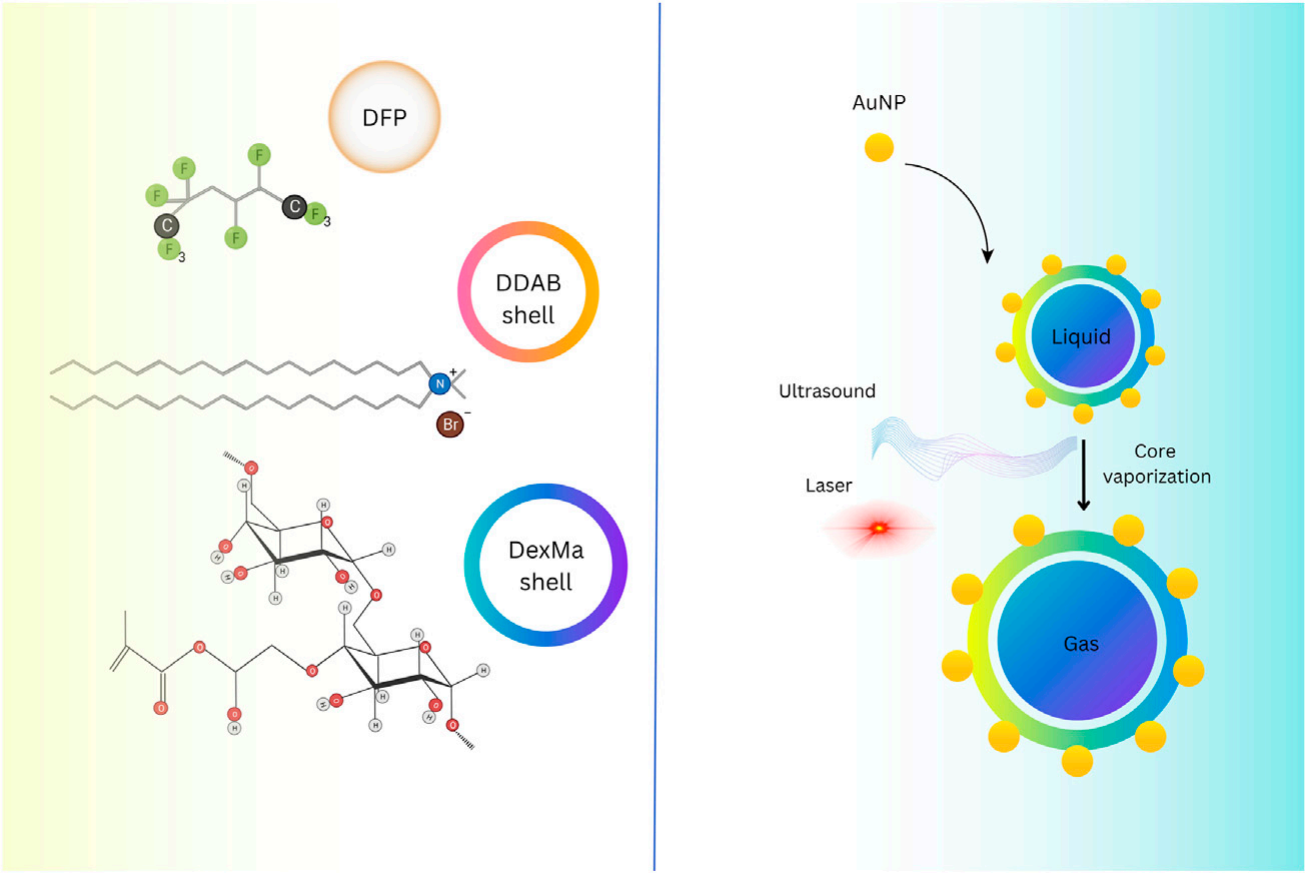
Hybrid-shelled perfluorocarbon microdroplets with elevated density and thin diameter dispersal (∼1 µm) operated in ultrasound- and laser-activated phase-change approach. Reprinted image. Reprinted with permission from Ref (Palmieri et al., 2022). Copyright (2022), Elsevier.

Ultrasound imaging relies on sound signals generated by the reflection or propagation of sound waves with frequencies above the audible range of humans (20 kHz<) (McCarthy et al., 2020). However, the NPs diameter should be 250 nm (Athanassiadis et al., 2022) (Figure 1). In ultrasound, these compounds are also utilized as Ligand-directed and Lipid-encapsulated agents. Because of the high surface area of these NPs, 50 to 500 ligands can be contained within them. These NPs are encapsulated with aptamers and polysaccharides (Figure 2) (Tran et al., 2007; Palmieri et al., 2022). This imaging method’s strengths include portability, adaptability, and usability.

Ultrasound contrast agents (USCAs) with a gas, liquid, and solid core generate elevated acoustic impedance contrasts within tissue interfaces, and they can generate the highest acoustic vehemence among the other classes. In terms of echogenicity and resilience, liquid-core USCAs have benefits over gas-core USCAs and disadvantages over solid-core USCAs. Liquid-core USCAs supply inadequate contrast enhancement due to their weak acoustic scattering inside the arteries as their inferior impedance. Liquid-based materials, such as PFC, including perfluoro-PFP, PFOB, and PFH, could accumulate in the target tissue and change phase from liquid to gas by devoting thermal energy, creating a unique echo in preclinical experiments (Tarighatnia et al., 2022). In ultrasound imaging by sulfur hexafluoride gas, if the diameter of the microbubbles is smaller than the diameter of the bubbles at room temperature (2.5 μm), it improves the passage of particles through the pulmonary capillaries. Size distribution and the particle size of PFCs have a strong influence on intravascular persistence and *in vivo* recognition. Using PFCs microbubbles with micrometric size increased the intravascular half-life, but elevating it will be difficult. The stabilization mechanism of bubbles got from perfluorocarbons is related to the mutual reaction of osmotic pressure and Laplace pressure, which delays the dissolution of bubbles in the blood (Tarighatnia et al., 2022).

By reducing the Ostwald coefficient, the stability of intravascular bubbles increases. The half-life of experimentally measured bubbles was always several orders of magnitude larger than the predicted values. In a study conducted in 2012 by Csongor Szijjarto et al. the size, size distribution, and stability characteristics of dimyristoylphosphatidylcholine (DMPC)-coated microbubbles on three PFH gas compounds (F-hexane), perfluoro diglyme (F-diglyme), and perfluoro triglyme (F-triglyme) were investigated. F-hexane, F-diglyme, and F-triglyme stabilized bubbles were half-lives 149 ± 8, 134 ± 3 and 76 ± 7 min, respectively. But the bubbles that do not contain PFCs gas have a half-life of only 34 ± 3 min, and these bubbles have a larger diameter and polydispersity. So, the size of microbubbles influences their half-life (Szíjjártó et al., 2012). The extravascular recognition potential of contrast agent microbubbles that are based on PFC and are commercially available is 10 times less compared to PFC emulsions that have a diameter of 100–200 nm.

Another imaging technique is photoacoustic imaging (PAI) or optoacoustic imaging. This technique is capable of imaging optical contrast with a penetration depth of several centimeters and an ultrasound resolution. The PAI method is based on thermoelastic effects. Exogenous or endogenous chromophores absorb light pulses; because light energy is transferred to heat, the rapid development of volume occurs, and eventually, sound waves are generated. PAI is used in various fields such as measuring oxygen saturation, angiogenesis, metastasis, breast imaging, and imaging specific tumor cell types using NPs. One of the widely used contrast agents in this technique is Phase-Shift (PS) PFC droplets or PFC emulsion, which is used in an encapsulated form. A study by Mangala Srinivas et al. used poly (D, L-lactic-*co*-glycolic acid) NPs loaded with perfluoro-15-crown-5-ether (PFCE) and ICG. This study aimed to investigate the photoacoustic effects of ICG. Due to the high sensitivity and penetration depth of the PAI method, this method was combined with FMRI. In this study, the optical absorption stability of PLGA-PFCE-ICG and ICG dye was studied by (Swider et al., 2018). Chen et al. (2020) designed a mitochondria-targeting liquid perfluorocarbon (PFC)-based oxygen delivery system for the synergistic photodynamic therapy (PDT)/photothermal therapy (PTT) of cancer *via* image guiding. Their novel approach accomplishes exceptional antitumor efficacy through an unprecedented structure with tumor mitochondria targeting, oxygen delivery, and synergistic PDT/PTT with dual-imaging direction.

### 2.1 PFCs to overcome hypoxia in PDT and RT techniques

Phototherapy, which includes two categories of photothermal therapy (PTT) and PDT, is a non-invasive procedure approved by the FDA. PDT comprises three main components: Photosensitizer, oxygen, and light (Goh et al., 2010). Efficient singlet oxygen is produced when a photosensitizer reacts with oxygen molecules under a specific wavelength of laser light. These singlet oxygens damage tumor cells and arteries by inducing cells to apoptosis, necrosis, and activation of immune responses. But with the salient advantages, you must also know some disadvantages (Xavierselvan et al., 2022).

When PS absorbs photons in the light, it changes from the base state to the transient state. This unstable state chooses one of two paths. It returns from the singlet state to the ground state by emitting fluorescence or energy loss. The second path changes from the singlet state to the long-lived triplet state through an intersection within the system (Hu et al., 2019). This triplet mode transfers energy to oxygen molecules through two mechanisms. Radical species are constructed by repositioning hydrogen or electrons when PS responds with organic molecules. Finally, radical species can react with oxygen to produce reactive oxygen species (ROS). In the second mechanism, the PSs in the excited state transfer energy directly to the oxygen molecules and lead to the production of activated singlet oxygen. These ROS oxidize subcellular organelles and destroy blood vessels (Wang et al., 2020). It eventually leads to light-induced cell death. In the PDT technique, the lifespan of singlet oxygen in PFCs is more extended than the cellular environment and water, which leads to the long-term effects of this technique. Radiotherapy (RT) uses ionizing radiation (X-ray, or γray) to generate free radicals, which damage DNA directly and indirectly to other cellular elements to induce DNA impairment and induce cancer cell death. Under hypoxia, these injuries heal immediately. This method is used in clinics to treat cancer. A little part of the energy of this radiation is fascinated by tumor cells. Most of this energy harms normal tissues; this method is non-specific. But oxygen molecules during radiotherapy forms radical peroxide that is more destructive and problematic, thus making it impossible for cell repair and DNA damage to stabilize. Cell damage by ionizing radiation depends to a large extent on the oxygen level of the cells. The environment around the tumor is hypoxic compared to healthy cells, so it is necessary to optimize it to increase the efficiency of this method. Liquid PFCs are widely used to optimize this treatment (Song et al., 2017). Table 2 lists several NPs combined with PFC to overcome the hypoxic conditions used in combination with photodynamic therapy and radiotherapy.

**TABLE 2:** Oxygen-carrying nanoparticles for tumor reoxygenation to enhance the antitumor.

Design	Combined treatment	Effectiveness	Ref
Physical dissolution of oxygen Lipid-stabilized PFC nanoemulsion and carbogen	RT	Carbogen alone decreased hypoxia levels substantially and conferred a smaller but not statistically significant survival advantage over and above radiation alone	Xiang et al. (2019)
LIP(IR780 and PFH)	PDT	tumor growth inhibition in Oxy-PDT mice treated with a low photosensitizer dosage and 20-s laser irradiation, whereas traditional PDT showed negligible tumour inhibition	Cheng et al. (2015)
polyethylene glycol (PEG) stabilized perfluorocarbon	RT	Improve tumor oxygenation and concentrate radiation energy in tumor regions to enhance the X-ray-induced DNA damages	Song et al. (2017)
(PFC) nano-droplets decorated with TaOx nanoparticles			
(TaOx@PFC-PEG)			
The PFC nanoliposomes (FI@Lip) and biocompatible NO donor S-nitrosated human serum albumin (HSA-SNO)	PDT	This combination strategy of FI@Lip and HSA-SNO obviously relieved intracellular hypoxia and decreased GSH to increase more toxic 1O_2_ generation for PDT enhancement	Alizadeh et al. (2019)
PFOB nanocapsules coated with PEGylated gold nanoshell	PDT	PGsP NCs could not only provide excellent contrast enhancement for dual modal ultrasound and CT imaging *in vitro* and *in vivo*, but also serve as effi cient photoabsorbers for photothermal ablation of tumors on xenografted nude mouse model	Ke et al. (2014)

Photodynamic therapy, which is one of the new methods in the treatment of cancer, whose anticancer effect is related to reactive oxygen species and singlet oxygen produced by oxygen in the photodynamic reaction. However, PDT turns off the vascular and consumes oxygen, In this condition, oxygen is less and hypoxia is intensified. After attaining superior anti-tumor therapy tracing the development of an effective approach to dominate a hypoxic tumor surrounding is highly desirable. Compared to other solvents, perfluorocarbons increase the half-life of singlet ^1^O_2_ by 10^5^ folds, so they are a very suitable carrier for oxygenation and overcoming hypoxic conditions (Fang et al., 2021).

### 2.2 PFC in cell tracking

Cell therapy approach used in treating various diseases, including cardiovascular disease, ischemia, type 1 diabetes, and cancer. Different stem cells and immunity are used for this purpose, but its tracing is essential to evaluate the function and position of the transplanted cell. Optic, ultrasound, MRI, CT, PET, and SPECT imaging modalities detect transplanted cells (Stanton et al., 2016). Different contrast agents are required depending on the technique.

PFCs are used in MRI and Ultra Sound imaging modalities. In the previous section, the importance of PFCs in imaging was discussed. Tracking is performed using the techniques discussed in the precautious section. But in this section, the different tissues to which the cell is attached and the role of PFCs as oxygen carriers are given in Table 3. These cells detect tumor antigens and have the ability to migrate to tissue and eventually penetrate tumor tissue. In an *in vitro* study, Gonzales et al. Labeled splenocyte and Ovalbumin T-cells with PFC and then examined them by FMRS/MRI. In an *in vivo* study, Gonzales and colleagues labeled splenocyte and ovalbumin T-cells in the liver, spleen, and lung with PFC and then examined them by FMRS/MRI. Finally, they concluded indivisible cells labeled with 19F are promising for FMRS/MRI-modality tracking. Therefore, labeling cells with PFC compounds such as perfluoropolyether (PFPE) is a promising way to monitor the treatment of cancer cells. FDA-approved PFC compounds used to label and track cells by FMRI are Cell Sense and V-Sense (Wu et al., 2020).

**TABLE 3:** Applications of^19^F MR in molecular imaging.

Type of PFC	Imaging purposes	Models	Ref
Perfluoropolyether	cell tracking	Dendritic cells	Ahrens et al. (2005)
PFOB and PFCE	cell tracking	stem/progenitor cells	Partlow et al. (2007)
PFCE	cell tracking	stem cells	Ruiz‐Cabello et al. (2008)
PFPE	cell tracking	antigen-specific T cells	Kadayakkara et al. (2010)
PFPE	stroke-damaged brain imaging	human neural stem cells (hNSCs)	Boehm-Sturm et al. (2014), Tennstaedt et al. (2015)
PFPE and PFOB	cellular imaging	glioma cells	Kislukhin et al. (2016)
PFCE	cell tracking and therapy	dendritic cells	Kislukhin et al. (2016)
PFCE	cardiac quantitative imaging	progenitor stem cells and macrophages	Constantinides et al. (2018)
PFTBA and PFD	anatomic distribution	mice	Mason et al. (1989)
PFTBA	organ biodistribution	rats	McGoron et al. (1994)
PFCE	molecular imaging of fibrin- targeted	*ex vivo* human samples	Morawski et al. (2004)
PFOB	tissue factor-targeted drug delivery	vascular smooth muscle cells	Zhou et al. (2009b)
PFOB	inflammation quantitative imaging	rats	Ahrens et al. (2011)
PFOB	ανβ3 integrin targeted	rabbits	
PFOC	intravascular oxygen tension evaluation	mice	Hu et al. (2013)
PFCE, PLGA	organ biodistribution	*ex vivo* human samples	Hoogendijk et al. (2020)
PFCE	inflammation quantification of intact tissue samples	*Ex vivo* mice sample	Díaz-López et al. (2010)
Perfluorohexane	cytotoxicity, hemolytic activity, biodistribution, biosafety, and antitumor activity	mice	Baghbani et al. (2017)

## 3 Therapeutic applications of PFCs in the treatment and diagnosis of diseases

### 3.1 Thrombosis

**FIGURE 3:**
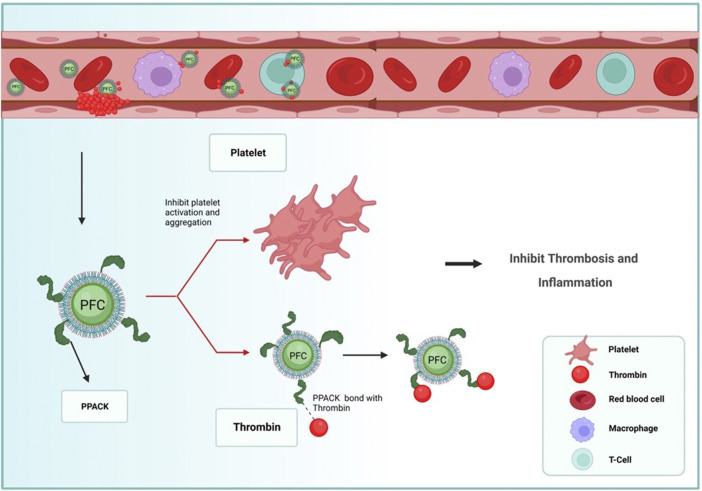
Schematic illustration of PFCs nanoparticles with an anti-coagulation cover (PPACK (Phe [D]-Pro-Arg-Chloromethylketone) that is related to the surface of PFC-core nanoparticle with a covalent bond (Myerson et al., 2010; Myerson et al., 2011). This arrangement acted as an adequate anticoagulant and prevented thrombosis locally in the damaged vessels as a thrombin leech to inhibit thrombosis and thrombin-activated inflammatory signaling.

Thrombus formation is critical in numerous cardiovascular disorders such as ischemic stroke, myocardial infarction, deep venous thrombosis, and pulmonary embolism, which are meaningful causes of morbidity and mortality worldwide (Stein et al., 2005; Taghizadeh et al., 2020). Also, Thrombus formation is the prime concern for using blood-contacting medical devices (Mauri et al., 2007; Mauri et al., 2014; Grover and Mackman, 2019; Virani et al., 2020). Percutaneous coronary intervention (PCI) and fibrinolysis with various anticoagulants and antiplatelet agents are the standard therapeutic procedures to prevent further clot progression field (Chattopadhyay et al., 2011; Olaf and Cooney, 2017). The restorative examples have limitations, including an almost short time window consistent with fierce regimens, thrombus formation still proceeding, and severe bleeding from using the systemically active anticoagulants (Osório, 2010; Robert, 2010; Siegel et al., 2022). Therefore, developing safer anticoagulants and a non-invasive treatment method is an ongoing pharmaceutical chase for managing thrombotic events in CVD Table 4 (Tran et al., 2007; Chen et al., 2021a; Wu et al., 2021). In this interest, PFC NPs, microbubbles composed of PFC combination with standard External low-frequency ultrasound (USD), define a medium technology with adjustable molecular imaging and provincial drug delivery in thrombosis events (Ravis et al., 1991; Flaim, 1994; Leese et al., 2000; Jacoby et al., 2014; Roberts et al., 2020; Manners et al., 2022). Myerson et al. (2010); Myerson et al. (2011) synthesized PPACK (Phe [D]-Pro-Arg-Chloromethylketone) and fastened these structures to the surface of PFC-core nanoparticle with the covalent bonds. These structures showed that the PPACK PFC nanoparticle could be an effective anticoagulant and prevent thrombosis, although the PPACK has not had these features alone. An increase in the number of PPACK ligands resulted in the maintenance of anticoagulants effects at the site of thrombosis and inhibition of activated thrombin event and inflammation (Figure 3). Bouvain and colleagues generate a non-invasive approach for explicit mapping of neutrophil dynamics by 19F-based MRI probes, using PFCs. *In-vivo* data showed this technique let to recognize undercover origins of inflammation in patients and also to separate cardiovascular disease circumstances on the point of extreme aggravation due to enriched neutrophil infiltration or activation (Bouvain et al., 2023). Liposomal bubbles (bubble liposome, BL) constructed of PFC gas and nano-sized liposomes covered by RGD sequence peptides on their exterior shell. These liposomal bubbles can connect to the glycoprotein IIb/IIIa complex. Mentioned complex duty is activating platelets which can improve the visualizing and accurate detection of exciting thrombus by conventional diagnostic ultrasound probes for thrombus imaging and disruption *in vitro and in vivo* (Hagisawa et al., 2013). Hagisawa et al. research indicated the fact that the reduction in speed of the clot with targeted liposomal bubbles was significantly more elevated than with non-targeted. Also, it proved that High-intensity USD orientation with targeted BL can acquire arterial recanalization in 90% of arteries, and the time to perfusion was quicker than the results for rt-PA therapy. Blood coagulation in Medical devices is one of the most challenging problems in designing these devices; the best approach for preventing coagulation is to use tethered liquid PFC (TLP) coating on the surface of instruments. The TLP bilayer coating decreases the adhesion of blood and prevents thrombus formation. Roberts et al. (2020) used TLP-coated (Tethered Liquid PFC) ECLS circuitry and immobilized-heparin on the surface; the coating was established for 6 h of circulation in swine and using no systemic heparin. The result is that TLP enables heparin-free ECLS for 6 h not to alter the membrane’s critical coagulation and does not affect gas exchange efficiency versus the clinical standard—immobilized heparin. Another challenging crisis is thrombus formation by conventional MRI and 1H MR angiography. These techniques may have an insignificant impact on blood flow. Biologically inert PFC nanoemulsions are used as 19F MRI, a unique technique for molecular imaging (Spuentrup et al., 2005a; Spuentrup et al., 2005b; Stoll et al., 2012; Guo et al., 2021). Temme et al. (2015) worked on new generating approaches in nan invasive for diagnosis with 1H/19F MRI acute deep venous thrombosis and pulmonary thromboembolic design targeted PFCs with sterol-based post-insertion technique (SPIT). This structure generates α2-antiplasmin–labeled PFCs (α2AP-PFCs) and allows the qualification of accomplished PFCs under favorable circumstances that sustain the functionality of labile ligands.

**TABLE 4:** Clinical application of PFCs.

Type of PFCs	Application	Disease	References
Dodecafluoropentane	Neuroprotection	Stroke	Culp et al. (2019)
Perfluorochemical plus O _2_	Whole-pancreas transplantation	Pancreas transplantation	Matsumoto et al. (2000)
Perfluorochemical plus O _2_	Pancreas transplantation	Pancreas transplantation	Toyama et al. (2003)
Perflubutane	Computed Tomography (CT) and Ultrasonography (US) imaging	Hyper vascular hepatocellular carcinoma	Numata et al. (2012)
Perfluorobutane microbubbles	US	colorectal liver metastases	Takahashi et al. (2012)
Perflubutane microbubble	US	focal liver lesions	Moriyasu and Itoh (2009)
liquid perfluorocarbon pads	3-T MRI	choice of optimal fat suppression method	Maehara et al. (2014)
Perflubutane microbubble	US	prostate cancer	Uemura et al. (2013)
Perfluorocarbon	fluorine-19 MRI	colorectal adenocarcinoma	Ahrens et al. (2014)
Perfluorocarbon	Following intratracheal (IT) delivery of PFC NP to locally deliver PFC NP in high concentrations into lung cancers	Lung cancer	Wu et al. (2018)

### 3.2 Rheumatoid arthritis

Rheumatoid arthritis (RA) is a chronic systemic inflammatory condition categorized as an autoimmune disease affecting approximately 1% of the global population (Giannini et al., 2020; Jahangir et al., 2022). RA is categorized as an autoimmune disease influencing approximately 1% of the global population. Disease manifestations are ongoing inflammation of synovial tissues, leading to articular cartilage and bone breakdown in the afflicted joints, and, in the long term, consequences are a significant functioning disability and actual death (Kourilovitch et al., 2014). The leading cause of this disease has not yet been defined. First, inflammation starts in synovium because of population increases of fibroblast-like synoviocytes (FLS) and macrophage-like synoviocytes (MLS), and hyperplasia in synovial tissue is showed. In the continuation of these changes, the synovial cells secreting metalloproteases (MMPs) and TNF-a trigger the growth of osteoclasts, which cause bone bruises (McInnes and Schett, 2007). All these events lead to chronic inflammation in RA by swelling and accumulation of recalling other inflammatory cells, such as macrophages and lymphocytes, and fibroblasts are activated (McInnes and O’Dell, 2010). Other factors that are involved in the inflammatory progress and irreversible damage to the cartilage in RA are TNF-a and IL-17; these factors have synergistic effects in boosting the production of IL-1, IL-6, and IL-8 and granulocyte territory stimulating factor (G-CSF) MMPs (McInnes and O’Dell, 2010; Chen et al., 2021b). Many aspects are involved in the onset of RA; for example, genetically predisposed people can suffer from this disease under the influence of environmental aspects such as bacterial or viral infection (Perricone et al., 2019).

Further environmental risk factors for RA include smoking, alcohol use, birth weight, breastfeeding, socioeconomic status, and ethnicity (Viatte et al., 2013; Paulissen et al., 2015; Baker et al., 2020; Ishikawa and Terao, 2020). Numerous imaging methods, for instance, MRI and computed tomography, diagnose RA patients. Early detection and prevention of further damage to the cartilage tissue is the most critical aspect of the treatment of RA; the main challenge in imaging procedures is the accurate diagnosis and quantified depth of damage in this disease to provide a practical guide to determine the exact dose of drug therapy (Wu et al., 2020) In line with the combination of imaging methods to achieve a good result researchers combined near-infrared (Sen Gupta, 2017) with 19f MRI used tagged NPs constructed from PLGA-PEG-Folate (Folate-NP), loaded with PFOB and indocyanine green (ICG) (Zhou et al., 2012; Vu-Quang et al., 2019). A common choice for RA patients to control symptoms establishes convergence with analgesics such as NSAIDs; combined regimens are usually preferred for achieving the best results and prolonged use (Svanström et al., 2018). Other choices in the remedy of RA can be mentioned as glucocorticoids (GCs), especially at the beginning of the treatment; this category of drugs in combination with co-therapy with other DMARDs is preferred. GCs show fast and effective outcomes, and because of the lower cost compared to other DMARDs, they are widespread. The main concern of GCs is high side effects in the long term, including an increased risk of cardiovascular disease, osteoporosis, infections, and altered glucose metabolism, which are usually not included in the patient’s medication regimen for a long time (Hardy and Cooper, 2018; Wu et al., 2021).

Methotrexate (MTX) is the standard DMARD treatment for RA. The mechanism of MTX is the inhibition of dihydrofolate reductase (DHFR). More contemporary methods of RA treatment, such as cytokine antagonists (TNF, IL-1, and IL-6 inhibitors or receptor antagonists), B-cell-depleting drugs, and T-cell disbursement modulators, can be mentioned. The most effective approach so far is particularly TNF inhibitors with MTX, which obtained responses from 60% to 70% of RA patients in the early stages of the disease. Nevertheless, high costs, the chance of spreading complications, and the loss or defeat to hold reaction over time are major problems in these approaches (Hayashi et al., 2020; Maciejewski et al., 2021).

As mentioned, handling the side effects of drugs is an important issue due to the chronicity of RA disease and compelling the patient to use therapy for a prolonged. The resolution is encapsulating the bioactive substances and modifying NPs for targeting the desired tissue, which leads to a reduction in the dosage and, ultimately, a reduction in side effects (Hoes et al., 2010) (Ye et al., 2008).

**FIGURE 4:**
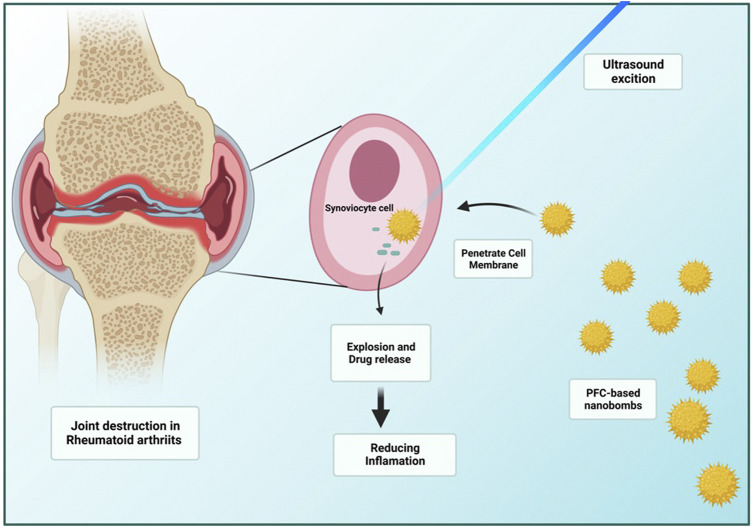
US-triggered perfluorocarbon (PFC)-based “nanobombs” for the targeted therapy of RA (Zhu et al., 2019). The targeted nanobombs structure includes thin-film hydration and a core of PFP-based nanodroplets (Maciejewski et al., 2021) loaded with glucocorticoid dexamethasone (Dex) and a shell of folic acid (FA)-grafted polyethylene glycol (PEG)-functionalized phospholipid (PFP-Dex@NDs-PEG-FA). The 1 MHz US is utilized as an initiator to activate the “explosion” of nanobombs and improve the drug departure as an efficient, targeted mechanism for RA therapy.

PFC nanodroplets with low boiling temperatures are now widely employed in ultrasonic medication delivery. Besides PFC biocompatibility and biodegradability, surface functionalizing with molecules such as PEG can boost the circulation period. The medication enclosed in the droplets can be given passively through increased permeability and retention (EPR) (Astafyeva et al., 2015). Zhu et al. (2019) synthesized folate and PEG-modified PFP-based nanodroplets loaded with Dexamethasone. For *in-vivo* testing, collagen-induced arthritis (CIA) SD rat model was developed. The *in vitro* drug release of “nanobombs” and contrast-enhanced US imaging were comprehensively studied. Targeting and cell viability of triggered macrophages were then tested, as shown in (Figure 4). The US expands the passive target through the EPR effect and discharges more drugs by eliminating the nanodroplets and the result indicated extraordinary inhibition of synovitis and joint collapse by declining the level of pro-inflammatory cytokines, acting as an effective targeted drug for RA therapy (Zhang et al., 2018). Anti-angiogenic fumagillin, a mycotoxin produced by Aspergillus fumagatus, inhibits the MMP2; Zhou et al. (2009a) demonstrated that v3-targeted PFC NPs administered systemically accumulated to the inflamed joints and quashed inflammatory arthritis.

In another work, Zhou and colleagues revealed that in a mouse model of arthritis, a single dosage of fumagillin-PFC NPs was injected systemically and synergized with the customary DMARD MTX to give numerous anti-inflammatory advantages with an adequate safety profile (Zhou et al., 2010). Zhou et al. (2012) utilize a lipase-labile (Sn 2) fumagillin prodrug associated with a lipid surface-to-surface targeted delivery mechanism. Dissolved fumagillin comparative to the PFC core and lipid-gadolinium conjugates *in vivo* to ease drug delivery and early drug release and overcome the inherent photo-instability of fumagillin.

Tang et al. (2017) nanoscale PLGA drug delivery system encapsulated oxygen-saturated PFP and IC. Tang’s study examined the cytotoxic effects of OI-NP–mediated PSDT against FLSs *in vitro*. Data showed that the OI-NPs were a steady and efficient carrier for delivering oxygen and indocyanine green, and the NPs increased cellular absorption in MH7A cells. In addition, MH7A cells treated with PSDT indicated an increase in the appearance of intracellular ROS. Pretreatment with the ROS scavenger N-acetylcysteine reversed the OI-NP–mediated PSDT–induced cell survival decrease.

### 3.3 Muscular dystrophies

*Muscular dystrophies* are disorders that show symptoms of dystrophic pathologic characteristics on muscles. Dystrophinopathies affect 1 in 5,000 to 1 in 6,000 live male births worldwide. Clinically cause, progressive weakness and defeat of muscle mass, and substantial mutability exist in the genetic and biochemical points. All these aspects result in a commonness of muscular dysfunction and respiratory and cardiac compromise, and the eyes and central nervous system may be under effect, too. Cognitive impairment, learning difficulties, and behavioral problems were also demonstrated later (Dongsheng et al., 2021). The manifestations of muscular dystrophies can be different in further people; various factors, such as genetic differences, affect the time of onset and severity of disorder complications. Current treatment for muscular dystrophy mainly comprises controlling the symptoms and reducing the patient’s crises, such as using corticosteroids. Of course, treatments based on genetic modification are under research, which hopes to restore the lost function in these patients. However, picking the proper treatment is still a challenge in the Muscular dystrophies (Carter et al., 2018; Pennati et al., 2021).

PFCs NPs are expressed as drug delivery carriers for muscular dystrophy therapeutic substitutes. As an outcome, this approach can enable reducing some expected adverse effects, such as toxicity in the long term. The PFC particles limit drug uptake in normal tissues and can target the desired area in muscular dystrophies. Research has investigated insufficient autophagy in mdx mice (Duchenne muscular dystrophy model) treated using PFC NPs loaded with rapamycin. Structure induces growth in skeletal muscle strength over the extent of cardiac contractile rendition (Bibee et al., 2014).

### 3.4 Cancer

**FIGURE 5:**
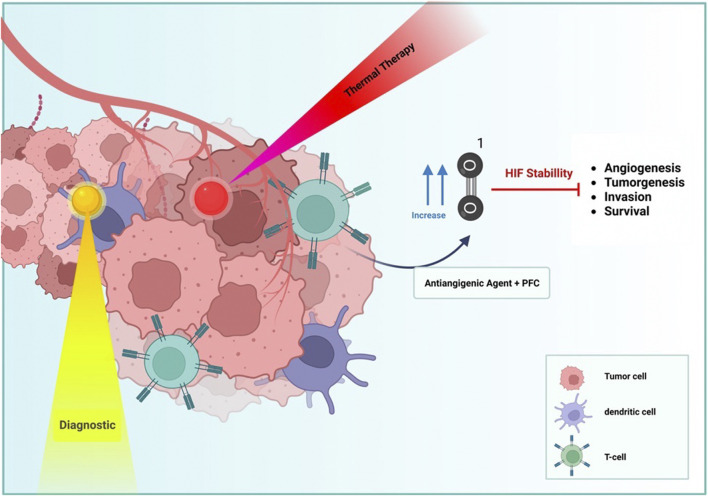
PFC NDs and NPs utilized different approaches that can present cancer therapy, such as diagnostic (yellow), thermal therapy (Red), and vascular distribution (Blue) by hypoxia effects.

There are numerous treatment options for solid tumor cancers. Among these techniques are pharmacological and chemotherapy approaches (Figure 5). Typically, cancer-specific drugs operate at the molecular level, specifically targeting a specific mutation. Nevertheless, it has been observed that cancer cells can evade the effects of drugs by completing a shortcut (Derakhshankhah et al., 2017). Therefore, chemotherapeutic methods are required to treat the disease. Radiotherapy and photodynamic therapy are included among the chemotherapy methods (Song et al., 2017; Hu et al., 2019). Although these strategies independently are not significantly adequate because part of the radiation is fascinated by healthy cells and the lack of oxygen in the tumor tissue, which is caused by inequality between oxygen supply and consumption due to the tumor’s rapid acceleration of the process of tumor growth. Tumors adapt their metabolism to oxygen-dependent microenvironments by activating hypoxia-inducing factors (HIF-1α) (Wang et al., 2020), which produce energy through an anaerobic process; hypoxic microenvironments regulate tumor growth and survival (Sun et al., 2020). Low oxygen levels in cells may be one of the primary causes of the uncontrolled growth of tumor cells in certain forms of cancer. Tissue oxygen deficiency is only a factor in the progression and development of cancerous masses; it is not the driving force. As the tumor’s oxygen level decreases, the tumor’s hypoxia worsens (Wang et al., 2020). Tumor cells express HIF-1 to survive in hypoxia. As a result of the genomic instability, altered tumor cell metabolism increased angiogenesis, and induced local immunosuppressive microenvironment caused by hypoxia, cancer cells become resistant to therapies. Two general strategies for overcoming tumor hypoxia are to deliver oxygen to the tumor site as a therapeutic agent and to take advantage of the unique environmental conditions that solid tumors have for targeted treatment.

Therefore, the oxygen concentration affects the efficacy of chemotherapy, photodynamic therapy, and radiotherapy. To have a toxic effect on cancer cells, this oxygen must exist in the singlet state. With the assistance of photodynamic therapy and radiotherapy, tissue oxygen can be converted to ROS. Oxygen-producing compounds and oxygen-carrying molecules can increase the oxygen concentration in tumor tissue to concentrate the effect of radiation on tumor tissue and overcome tissue hypoxia conditions, eventually leading to the death of more tumor cells. The article focuses on oxygen-carrying compounds, with PFCs being the most important. Zhou et al. (2019a). designed PFC and etoposide (EP) loaded porous hollow Fe_3_O_4_–based theranostic nano platform qualified of delivering oxygen to solid tumors to improve their vulnerability against EP. Outcomes showed that oxygen could be released at an average rate from the porous hollow magnetic Fe_3_O_4_ nanoparticles (PHMNPs) over a vast period, decreasing the hypoxia-induced EP resistance of tumor cells. Kim et al. created a drug delivery transport by exposure to near-infrared (NIR) light for drug release and tumor therapy. designed prepared based on a thin film method and utilizing Melanin, perfluorohexane (PFH), and 5-fluorouracil (5-FU)-loaded liposomes (melanin@PFH@5-FU-liposomes). The result indicated that tumor growth was virtually inhibited by the injection of melanin@PFH@5-FU-liposomes with laser irradiation (Kim and Lee, 2022).

Temperature and pH do not affect the oxygen-carrying capacity of PFCs. These PFC-based NPs have recently become famous for oxygenating tumors and enhancing the effects of anticancer treatments. PFC fluids such as PFTBA, PFP, PFH, PFOB, and others have been used as oxygen carriers for tumor hypoxia. These oxygen-carrying compounds are stabilized with lipids, polymers, and proteins because they are insoluble in water. With the immiscibility of oxygen in water, PFCs are emulsified with surfactants to serve as oxygen carriers. The surfactants Poloxamer F68 and Poloxamer 188 are two of these compounds.

PFC nanoemulsions are utilized in both liquid NPs and gas bubble forms. Nanoemulsions droplets, when evaporated, produce microbubbles and increase oxygen delivery to the tumor. In mice with pancreatic tumors, the partial pressure of tumor O_2_ rises by up to 400% when small doses of F-Pentan Phase-Shift nanoemulsions (P-SNE) are used. However, this increase in oxygen pressure occurs under conditions that combine with carbogen or radiation; Under these conditions, the tumor volume decreases significantly (Krafft, 2020).

^19^FMRI method is very effective for quantitatively evaluating O_2_ gas pressure around tumor tissues. Non-invasive methods produce O_2_ gas pressure surrounding tumor tissue and recreate a guiding part in cancer treatment. With the assistance of the FMRI method, the pO_2_ level of tumor tissue can be measured before and after oxygenation, in which case it is possible to control the tumor response appropriately to oxygenation. Because FMRI-relate because spin-network R1 relaxation rate is susceptible to pO_2_. PFC emulsions can be injected intravenously or into the tumor and t, then the pO_2_ of the tumor can be measured. Studies have indicated that tumor hypoxia is directly related to its size (Wu et al., 2020). Inspiring work done by Yang and colleagues designed an osimertinib-loaded perfluoro-15-crown-5-ether (AZD9291-PFCE) nanoemulsions, through intratracheal and intravenous delivery, synergizes with 119F MRI-guided low-intensity focused ultrasound (LIFU) for lung cancer therapy. Pulmonary delivery of AZD9291-PFCE nanoemulsions in orthotopic lung carcinoma models performs immediate diffusion of the nanoemulsions in lung tissues and tumors without side effects. Likewise, LIFU triggered drug release from the AZD9291-PFCE nanoemulsions and particularly boosts tumor vascular and tumor tissue permeability. The result showed validation of the treatment effect of AZD9291-PFCE nanoemulsions in resected human lung cancer tissues, confirming the translational prospect to enrich clinical outputs of the lung cancer therapy (Yang et al., 2022a).

If we examine perfluorocarbons in terms of safety in diagnosing and treating diseases, among various perfluorocarbon compounds, compounds such as perfluorooctanoic acid (PFOA) and perfluorooctane sulfonic acid (PFOS) increase the risk of cardiovascular diseases compared to other PFC compounds. Compounds such as oxygenate and oxofluor were subjected to many safety tests, and although their results have not been published in scientific texts, they received a license for clinical use due to their safety (Spahn, 1999). The contrast agent Gd or iodate and PFC NPs can be utilized in molecular imaging. In a comparison between them, which was done to check the injury and function of kidney patients, the results indicated that NP PFCs do not have kidney toxicity and 24 h after consumption, the profile of them have good safety and no toxicity has been reported in human and animal samples (Chen et al., 2013). Table 4 include some clinical studies based on PFCs application in cancer treatment and diagnosis.

## 4 Limitations and challenges

Microbubbles take center stage in ultrasound imaging and therapy because of their sharp disparity and therapeutic efficiency. Regardless, stability limitations yielded by the diffusion of the core gas across the shell still exist. To overcome this limitation, use mixed materials, such as PEG, for shells or blend core gases, such as nitrogen and PFCs. Still, PFC NPs as distinction agents will also incur additional costs. Enhanced shell resilience can improve microbubble functionality and *in vivo* therapeutic application strategies. Another limitation is the size used in drug delivery to target tissues (Chen et al., 2013). The micrometric ratios of microbubbles restrict their capability to penetrate *via* intercellular confluence.

Therefore, most microbubble applications are limited to blood vessels. Studies tracking the pharmacokinetics in whole blood by mass spectroscopy and the PFOB core used in gas chromatography occasionally demonstrated substantial loss of the active compound during circulation before reaching the neovascular target quickly (Zhou et al., 2012).

PFCs have demonstrated promise in delivering oxygen and therapeutic agents to cancer cells, their delivery can also be non-specific, leading to potential off-target effects. Additional research is a must to generate methods for targeting PFCs specifically in diseased cells and tissues. Although PFCs have shown low toxicity in preclinical studies (Lehmler, 2008; Zhou et al., 2019b), there is still restricted data on their long-term safety and possible side effects. Additionally, research is required to fully comprehend the safety profile of PFCs, specifically in the context of repeated or prolonged exposure (Tak and Barraclough, 2018; Wikström et al., 2019; Szilagyi et al., 2020). A further issue is PFC’s cost, which can be expensive to produce and purify, and this cost may limit their overall use as a therapeutic agent.

Correspondingly, PFCs are foreign substances to the body, and it is conceivable that they could elicit an immune response, potentially diminishing their effectiveness over time. More research is ought to understand the potential for an immune response to PFCs and to acquire techniques for minimizing it (Zhou et al., 2019b; Moasefi et al., 2021; Yang et al., 2022b). Several factors for a successful outcome are needed for using PFCs in different theranostic applications, including the right dose for use in each application. Hill study on different clinical trials for investigation failure of PFC due to dose restriction, duration of demanded therapy, and the possible impact of extreme hemodilution on neurocognitive decline dose (Hill, 2019). Lim et al. (2000) investigated an *in-vivo* model of acute lung injury, and hypothesized that there was an optimal dose of PFC for PLV (around 9 mL/kg). Data showed that the rabbit worsened at high doses of PFC (≥12 mL/kg). Behind earlier progress, Cst plateaued at and beyond the 9 mL/kg dose. Expansions in airway pressure at the high-dose content were due to the accumulation of both elastic and resistive components.

## 5 Conclusion and future perspectives

Here, we concentrated on the applications of PFCs in molecular imaging, cell tracking, therapeutic drug delivery, and monitoring therapy efficacy. Promotions and progress in PFCs oxygen carrier, treatments or imaging such as PFC encapsulation in red blood cell membranes, nanodroplet, and nanoemulsions are promising; for example, PFC nanoemulsions are multifunctional agents competent for imaging in different approaches such as MRI, PAI as ultrasound platforms. However, comprehensive assurance in translational models is demanded before clinical usage. As in various circumstances, the imaging ability and safety of PFC are desirable but incompatible; Designating standards in the formulation of PFC nanoemulsions is an achievable near-term goal. Clinical translation of imaging technologies using PFC should be carefully examined, and long-term toxicity problems should be investigated. Desiring to generate biocompatible and high capacity artificial oxygen carriers conducts to safe PFCs formulations and are still evolving new applications and vowing new formulations. Another aspect of using PFCs is molecular imaging; PFCs NPs are relatively bio-inert and have a long-term systematic half-life, which permits indelible binding to ligands such as polyethylene glycol (PEG), which can increase circulation duration, makes PFCs suitable for widespread use in MRI techniques, photoacoustic imaging (PAI) with a penetration depth of several centimeters and ultrasound resolution. Early and depth detection allows for prematurely diagnosing multiple diseases such as thrombosis, rheumatoid arthritis, and cancer with high explicitness. Also, PFCs NPs are employed in ultrasound and MRI to label cells, target distinct epitopes in the tumor, monitor treatment effectiveness, quantify tumor characteristics, and detect changes in the tumor’s surrounding environment. In the outlook of tumor restriction, oxygen-producing compounds, and oxygen-carrying molecules can increase the oxygen concentration in tumor tissue to thicken the effect of radiation on tumor tissues; this approach crushes tissue hypoxia conditions, eventually leading to the death of more tumor cells, PFCs can promote these features and temperature and pH do not affect the oxygen-carrying capacity of PFCs. More discussed: While PFCs have shown promise in delivering oxygen and therapeutic agents to cancer cells, there is even space for advancement in terms of the specificity and efficiency of targeting. Further research is needed to design new approaches for orchestrating PFCs to specific cells and tissues and optimize their capability to deliver therapeutic agents. The safety of PFCs as a therapeutic agent has yet to be fully established. Further studies are required to apprehend the long-term outcomes of PFCs on the body, including potential toxicities and side effects. PFCs have been shown to enrich the efficacy of other cancer treatments, such as radiation therapy and chemotherapy. Further research is needed to specify the optimal combination of PFCs with these and other therapies and determine the most effective dosing strategies. While PFCs have been studied largely in the context of cancer treatment, they may have potential applications in other areas, such as tissue engineering, wound healing, and blood alternate. Additional research is needed to explore these possibilities and to determine the most effective ways to utilize PFCs in these contexts. In conclusion, the use of PFCs as a theranostic agent is a promising area of research with a lot of potential for future growth. However, more research is needed to thoroughly understand their safety and efficacy and to pinpoint the most effective ways to utilize PFCs in treating diseases.

## References

[B1] Abutarboush R. Saha B. K. Mullah S. H. Arnaud F. G. Haque A. Aligbe C. (2016). Cerebral microvascular and systemic effects following intravenous administration of the perfluorocarbon emulsion perftoran. J. Funct. Biomaterials 7 (4), 29. 10.3390/jfb7040029 PMC5197988 27869709

[B2] Ahrens E. T. Flores R. Xu H. Morel P. A. (2005). *In vivo* imaging platform for tracking immunotherapeutic cells. Nat. Biotechnol. 23 (8), 983–987. 10.1038/nbt1121 16041364

[B3] Ahrens E. T. Helfer B. M. O'Hanlon C. F. Schirda C. (2014). Clinical cell therapy imaging using a perfluorocarbon tracer and fluorine‐19 MRI. Magnetic Reson. Med. 72 (6), 1696–1701. 10.1002/mrm.25454 PMC4253123 25241945

[B4] Ahrens E. T. Young W-B. Xu H. Pusateri L. K. (2011). Rapid quantification of inflammation in tissue samples using perfluorocarbon emulsion and fluorine-19 nuclear magnetic resonance. Biotechniques 50 (4), 229–234. 10.2144/000113652 21548906 PMC5012185

[B5] Ahrens E. T. Zhong J. (2013). *In vivo*MRI cell tracking using perfluorocarbon probes and fluorine-19 detection. NMR Biomed. 26 (7), 860–871. 10.1002/nbm.2948 23606473 PMC3893103

[B6] Alayash A. I. (2014). Blood substitutes: Why haven’t we been more successful? Trends Biotechnol. 32 (4), 177–185. 10.1016/j.tibtech.2014.02.006 24630491 PMC4418436

[B7] Alizadeh S. Irani S. Bolhassani A. Sadat S. M. (2019). HR9: An important cell penetrating peptide for delivery of HCV NS3 DNA into HEK-293t cells. Avicenna J. Med. Biotechnol. 12 (1), 44–51. PMC7035460 32153738

[B8] Astafyeva K. Somaglino L. Desgranges S. Berti R. Patinote C. Langevin D. (2015). Perfluorocarbon nanodroplets stabilized by fluorinated surfactants: Characterization and potentiality as theranostic agents. J. Mater Chem. B 3 (14), 2892–2907. 10.1039/c4tb01578a 32262418

[B9] Athanassiadis A. G. Ma Z. Moreno-Gomez N. Melde K. Choi E. Goyal R. (2022). Ultrasound-Responsive systems as components for smart materials. Chem. Rev. 122 (5), 5165–5208. 10.1021/acs.chemrev.1c00622 34767350 PMC8915171

[B10] Baghbani F. Chegeni M. Moztarzadeh F. Mohandesi J. A. Mokhtari-Dizaji M. (2017). Ultrasonic nanotherapy of breast cancer using novel ultrasound-responsive alginate-shelled perfluorohexane nanodroplets: *In vitro* and *in vivo* evaluation. Mater. Sci. Eng. C 77, 698–707. 10.1016/j.msec.2017.02.017 28532082

[B11] Baker J. F. England B. R. Mikuls T. R. Hsu J. Y. George M. D. Pedro S. (2020). Changes in alcohol use and associations with disease activity, health status, and mortality in rheumatoid arthritis. Arthritis care and Res. 72 (3), 301–308. 10.1002/acr.23847 PMC6754326 30891938

[B12] Barnett B. P. Ruiz-Cabello J. Hota P. Liddell R. Walczak P. Howland V. (2011). Fluorocapsules for improved function, immunoprotection, and visualization of cellular therapeutics with MR, US, and CT imaging. Radiology 258 (1), 182–191. 10.1148/radiol.10092339 20971778 PMC3009379

[B13] Bibee K. P. Cheng Y. J. Ching J. K. Marsh J. N. Li A. J. Keeling R. M. (2014). Rapamycin nanoparticles target defective autophagy in muscular dystrophy to enhance both strength and cardiac function. Faseb J. 28 (5), 2047–2061. 10.1096/fj.13-237388 24500923 PMC3986846

[B14] Boehm-Sturm P. Aswendt M. Minassian A. Michalk S. Mengler L. Adamczak J. (2014). A multi-modality platform to image stem cell graft survival in the naive and stroke-damaged mouse brain. Biomaterials 35 (7), 2218–2226. 10.1016/j.biomaterials.2013.11.085 24355489

[B15] Bouchlaka M. N. Ludwig K. D. Gordon J. W. Kutz M. P. Bednarz B. P. Fain S. B. (2016). 19F-MRI for monitoring human NK cells *in vivo* . Oncoimmunology 5 (5), e1143996. 10.1080/2162402x.2016.1143996 27467963 PMC4910731

[B16] Bouvain P. Ding Z. Kadir S. Kleimann P. Kluge N. Tiren Z-B. (2023). Non-invasive mapping of systemic neutrophil dynamics upon cardiovascular injury. Nat. Cardiovasc. Res. 2, 126–143. 10.1038/s44161-022-00210-w PMC11357992 39196054

[B17] Carter J. C. Sheehan D. W. Prochoroff A. Birnkrant D. J. (2018). Muscular dystrophies. Clin. Chest Med. 39 (2), 377–389. 10.1016/j.ccm.2018.01.004 29779596

[B18] Charbe N. B. Castillo F. Tambuwala M. M. Prasher P. Chellappan D. K. Carreño A. (2022). A new era in oxygen therapeutics? From perfluorocarbon systems to haemoglobin-based oxygen carriers. Blood Rev. 54, 100927. 10.1016/j.blre.2022.100927 35094845

[B19] Chattopadhyay D. Al Samaraee A. Bhattacharya V. (2011). An update on the management and treatment of deep vein thrombosis. Cardiovasc Hematol. Agents Med. Chem. 9 (4), 207–217. 10.2174/187152511798120921 22150031

[B20] Chen J. Cheng W. Li J. Wang Y. Chen J. Shen X. (2021). Notch‐1 and notch‐3 mediate hypoxia‐induced activation of synovial fibroblasts in rheumatoid arthritis. Arthritis and Rheumatology. 73 (10), 1810–1819. 10.1002/art.41748 33844448

[B21] Chen J. Pan H. Lanza G. M. Wickline S. A. (2013). Perfluorocarbon nanoparticles for physiological and molecular imaging and therapy. Adv. chronic kidney Dis. 20 (6), 466–478. 10.1053/j.ackd.2013.08.004 24206599 PMC4074885

[B22] Chen J. Zhang X. Millican R. Sherwood J. Martin S. Jo H. (2021). Recent advances in nanomaterials for therapy and diagnosis for atherosclerosis. Adv. Drug Deliv. Rev. 170, 142–199. 10.1016/j.addr.2021.01.005 33428994 PMC7981266

[B23] Chen S. Huang B. Pei W. Wang L. Xu Y. Niu C. (2020). <p&gt;Mitochondria-Targeting oxygen-sufficient perfluorocarbon nanoparticles for imaging-guided tumor phototherapy</p&gt;. Int. J. Nanomedicine 15, 8641–8658. 10.2147/ijn.s281649 33177823 PMC7652575

[B24] Cheng Y. Cheng H. Jiang C. Qiu X. Wang K. Huan W. (2015). Perfluorocarbon nanoparticles enhance reactive oxygen levels and tumour growth inhibition in photodynamic therapy. Nat. Commun. 6 (1), 8785–8788. 10.1038/ncomms9785 26525216 PMC4659941

[B25] Constantinides C. Maguire M. McNeill E. Carnicer R. Swider E. Srinivas M. (2018). Fast, quantitative, murine cardiac 19F MRI/MRS of PFCE-labeled progenitor stem cells and macrophages at 9.4 T. PLoS One 13 (1), e0190558. 10.1371/journal.pone.0190558 29324754 PMC5764257

[B26] Cosco D. Fattal E. Fresta M. Tsapis N. (2015). Perfluorocarbon-loaded micro and nanosystems for medical imaging: A state of the art. J. Fluor. Chem. 171, 18–26. 10.1016/j.jfluchem.2014.10.013

[B27] Culp W. C. Onteddu S. S. Brown A. Nalleballe K. Sharma R. Skinner R. D. (2019). Dodecafluoropentane emulsion in acute ischemic stroke: A phase ib/II randomized and controlled dose-escalation trial. J. Vasc. Interv. Radiol. 30 (8), 1244–1250.e1. 10.1016/j.jvir.2019.04.020 31349978

[B28] Derakhshankhah H. Izadi Z. Alaei L. Lotfabadi A. Saboury A. A. Dinarvand R. (2017). Colon cancer and specific ways to deliver drugs to the large intestine. Anti-Cancer Agents Med. Chem. Former. Curr. Med. Chemistry-Anti-Cancer Agents) 17 (10), 1317–1327. 10.2174/1871520617666170213142030 28270073

[B29] Díaz-López R. Tsapis N. Fattal E. (2010). Liquid perfluorocarbons as contrast agents for ultrasonography and 19F-MRI. Pharm. Res. 27 (1), 1–16. 10.1007/s11095-009-0001-5 19902338

[B30] Dongsheng D Nathalie G Shin'ichi T (2021), Duchenne muscular dystrophy. Nat. Rev. Dis. Prim 7(1):14. 33602922 10.1038/s41572-021-00255-4

[B31] Fang H. Gai Y. Wang S. Liu Q. Zhang X. Ye M. (2021). Biomimetic oxygen delivery nanoparticles for enhancing photodynamic therapy in triple-negative breast cancer. J. nanobiotechnology 19 (1), 81–14. 10.1186/s12951-021-00827-2 33743740 PMC7981819

[B32] Flaim S. F. (1994). Pharmacokinetics and side effects of perfluorocarbon-based blood substitutes. Artif. Cells, Blood Substitutes, Biotechnol. 22 (4), 1043–1054. 10.3109/10731199409138801 7849908

[B33] Giannini D. Antonucci M. Petrelli F. Bilia S. Alunno A. Puxeddu I. (2020). One year in review 2020: Pathogenesis of rheumatoid arthritis. Clin. Exp. Rheumatol. 38 (3), 387–397. 10.55563/clinexprheumatol/3uj1ng 32324123

[B34] Goh F. Gross J. D. Simpson N. E. Sambanis A. (2010). Limited beneficial effects of perfluorocarbon emulsions on encapsulated cells in culture: Experimental and modeling studies. J. Biotechnol. 150 (2), 232–239. 10.1016/j.jbiotec.2010.08.013 20804794 PMC2962717

[B35] Grover S. P. Mackman N. (2019). Intrinsic pathway of coagulation and thrombosis: Insights from animal models. Arteriosclerosis, Thrombosis, Vasc. Biol. 39 (3), 331–338. 10.1161/atvbaha.118.312130 PMC12578993 30700128

[B36] Guo B. Li Z. Tu P. Tang H. Tu Y. (2021). Molecular imaging and non-molecular imaging of atherosclerotic plaque thrombosis. Front. Cardiovasc Med. 8, 692915. 10.3389/fcvm.2021.692915 34291095 PMC8286992

[B37] Hagisawa K. Nishioka T. Suzuki R. Maruyama K. Takase B. Ishihara M. (2013). Thrombus‐targeted perfluorocarbon‐containing liposomal bubbles for enhancement of ultrasonic thrombolysis: *In vitro* and *in vivo* study. J. Thrombosis Haemostasis 11 (8), 1565–1573. 10.1111/jth.12321 23773778

[B38] Hardy R. Cooper M. S. (2018). Unravelling how glucocorticoids work in rheumatoid arthritis. Nat. Rev. Rheumatol. 14 (10), 566–567. 10.1038/s41584-018-0079-4 30181582

[B39] Hayashi K. Sada K. E. Asano Y. Asano S. H. Yamamura Y. Ohashi K. (2020). Risk of higher dose methotrexate for renal impairment in patients with rheumatoid arthritis. Sci. Rep. 10 (1), 18715. 10.1038/s41598-020-75655-9 33127957 PMC7599222

[B40] Hill S. E. (2019). Perfluorocarbons: Knowledge gained from clinical trials. Shock 52 (1S), 60–64. 10.1097/shk.0000000000001045 29087985

[B41] Hoes J. N. Jacobs J. W. Buttgereit F. Bijlsma J. W. (2010). Current view of glucocorticoid co-therapy with DMARDs in rheumatoid arthritis. Nat. Rev. Rheumatol. 6 (12), 693–702. 10.1038/nrrheum.2010.179 21119718

[B42] Hoogendijk E. Swider E. Staal A. H. White P. B. van Riessen N. K. Glaßer G. (2020). Continuous-Flow production of perfluorocarbon-loaded polymeric nanoparticles: From the bench to clinic. ACS Appl. Mater. interfaces 12 (44), 49335–49345. 10.1021/acsami.0c12020 33086007 PMC7645868

[B43] Hu H. Yan X. Wang H. Tanaka J. Wang M. You W. (2019). Perfluorocarbon-based O 2 nanocarrier for efficient photodynamic therapy. J. Mater. Chem. B 7 (7), 1116–1123. 10.1039/c8tb01844h 32254779

[B44] Hu L. Chen J. Yang X. Caruthers S. D. Lanza G. M. Wickline S. A. (2013). Rapid quantification of oxygen tension in blood flow with a fluorine nanoparticle reporter and a novel blood flow‐enhanced‐saturation‐recovery sequence. Magnetic Reson. Med. 70 (1), 176–183. 10.1002/mrm.24436 PMC3873607 22915328

[B45] Ishikawa Y. Terao C. (2020). The impact of cigarette smoking on risk of rheumatoid arthritis: A narrative review. Cells 9 (2), 475. 10.3390/cells9020475 32092988 PMC7072747

[B46] Jacoby C. Temme S. Mayenfels F. Benoit N. Krafft M. P. Schubert R. (2014). Probing different perfluorocarbons for *in vivo* inflammation imaging by 19F MRI: Image reconstruction, biological half‐lives and sensitivity. NMR Biomed. 27 (3), 261–271. 10.1002/nbm.3059 24353148

[B47] Jägers J. Wrobeln A. Ferenz K. B. (2020). Perfluorocarbon-based oxygen carriers: From physics to physiology. Pflügers Archiv-European J. Physiology 473, 139–150. 10.1007/s00424-020-02482-2 33141239 PMC7607370

[B48] Jahangir S. Zeydabadinejad S. Izadi Z. Habibi-Anbouhi M. Hajizadeh-Saffar E. (2022). “New advanced therapy medicinal products in treatment of autoimmune diseases,” in Translational autoimmunity (Elsevier), 319–359.

[B49] Kadayakkara D. K. Janjic J. M. Pusateri L. K. Young W. B. Ahrens E. T. (2010). *In vivo* observation of intracellular oximetry in perfluorocarbon‐labeled glioma cells and chemotherapeutic response in the CNS using fluorine‐19 MRI. Magnetic Reson. Med. 64 (5), 1252–1259. 10.1002/mrm.22506 PMC2965778 20860007

[B50] Ke H. Yue X. Wang J. Xing S. Zhang Q. Dai Z. (2014). Gold nanoshelled liquid perfluorocarbon nanocapsules for combined dual modal ultrasound/CT imaging and photothermal therapy of cancer. Small 10 (6), 1220–1227. 10.1002/smll.201302252 24500926

[B52] Kim M. A. Lee C-M. (2022). NIR-Mediated drug release and tumor theranostics using melanin-loaded liposomes. Biomaterials Res. 26 (1), 22. 10.1186/s40824-022-00270-w PMC9164422 35659113

[B53] Kislukhin A. A. Xu H. Adams S. R. Narsinh K. H. Tsien R. Y. Ahrens E. T. (2016). Paramagnetic fluorinated nanoemulsions for sensitive cellular fluorine-19 magnetic resonance imaging. Nat. Mater. 15 (6), 662–668. 10.1038/nmat4585 26974409 PMC5053764

[B54] Kourilovitch M. Galarza-Maldonado C. Ortiz-Prado E. (2014). Diagnosis and classification of rheumatoid arthritis. J. Autoimmun. 48, 26–30. 10.1016/j.jaut.2014.01.027 24568777

[B55] Krafft M. P. (2020). Alleviating tumor hypoxia with perfluorocarbon-based oxygen carriers. Curr. Opin. Pharmacol. 53, 117–125. 10.1016/j.coph.2020.08.010 32979727

[B56] Lambert E. Gorantla V. S. Janjic J. M. (2019). Pharmaceutical design and development of perfluorocarbon nanocolloids for oxygen delivery in regenerative medicine. Nanomedicine 14 (20), 2697–2712. 10.2217/nnm-2019-0260 31657273

[B57] Lauby R. S. Johnson S. A. Meledeo M. A. Bynum J. Schauer S. G. (2022). A scoping review of promising alternative blood products for prolonged field care. Med. J. US Army Medical Center of Excellence (MEDCoE). 35373321

[B58] Leese P. T. Noveck R. J. Shorr J. S. Woods C. M. Flaim K. E. Keipert P. E. (2000). Randomized safety studies of intravenous perflubron emulsion. I. Effects on coagulation function in healthy volunteers. Anesth. Analgesia. 91 (4), 804–811. 10.1097/00000539-200010000-00008 11004030

[B59] Lehmler H-J. (2008). Anti-inflammatory effects of perfluorocarbon compounds. Expert Rev. Respir. Med. 2 (2), 273–289. 10.1586/17476348.2.2.273 20477255

[B60] Li W. B. Cheng Y. Z. Yang D. H. Liu Y. W. Han B. H. (2022). Fluorine‐containing covalent organic frameworks: Synthesis and application. China: Macromolecular Rapid Communications, 2200778. 10.1002/marc.202200778 36404104

[B61] Lim C-M. Koh Y. Jung B. O. Lee S. D. Kim W. S. Kim D. S. (2000). An optimal dose of perfluorocarbon for respiratory mechanics in partial liquid ventilation for dependent lung-dominant acute lung injury. Chest 117 (1), 199–204. 10.1378/chest.117.1.199 10631220

[B62] Liu L. Gou D. Song Y. Li M. Gu J. Zhang Y. (2022). Perfluorocarbon restrains inflammation and cell apoptosis in rats with lung ischemia-reperfusion injury via down-regulation of TLR4/NF-κB signaling pathway. Trop. J. Pharm. Res. 21 (12), 2533–2539. 10.4314/tjpr.v21i12.5

[B63] Luo L. Chen Z. Gong T. Ye Q. Li H. Guo Y. (2023). Cytosolic perfluorocarbon delivery to platelets via albumin for antithrombotic therapy. J. Control. Release 355, 109–121. 10.1016/j.jconrel.2023.01.036 36682727

[B64] Maciejewski M. Sands C. Nair N. Ling S. Verstappen S. Hyrich K. (2021). Prediction of response of methotrexate in patients with rheumatoid arthritis using serum lipidomics. Sci. Rep. 11 (1), 7266. 10.1038/s41598-021-86729-7 33790392 PMC8012618

[B65] Maehara M. Ikeda K. Kurokawa H. Ohmura N. Ikeda S. Hirokawa Y. (2014). Diffusion-weighted echo-planar imaging of the head and neck using 3-T MRI: Investigation into the usefulness of liquid perfluorocarbon pads and choice of optimal fat suppression method. Magn. Reson. imaging 32 (5), 440–445. 10.1016/j.mri.2014.01.011 24582547

[B66] Manners N. Priya V. Mehata A. K. Rawat M. Mohan S. Makeen H. A. (2022). Theranostic nanomedicines for the treatment of cardiovascular and related diseases: Current strategies and future perspectives. Pharmaceuticals 15 (4), 441. 10.3390/ph15040441 35455438 PMC9029632

[B67] Mason R. P. Antich P. P. Babcock E. E. Gerberich J. L. Nunnally R. L. (1989). Perfluorocarbon imaging *in vivo*: A 19F MRI study in tumor-bearing mice. Magn. Reson. imaging 7 (5), 475–485. 10.1016/0730-725x(89)90402-5 2607898

[B68] Matsumoto S. Kandaswamy R. Sutherland D. E. R. Hassoun A. A. Hiraoka K. Sageshima J. (2000). Clinical application of the two-layer (university of Wisconsin solution/perfluorochemical plus O 2) method of pancreas preservation before transplantation. Transplantation 70 (5), 771–774. 10.1097/00007890-200009150-00010 11003355

[B69] Mauri L. Hsieh W. Massaro J. M. Ho K. K. D'Agostino R. Cutlip D. E. (2007). Stent thrombosis in randomized clinical trials of drug-eluting stents. N. Engl. J. Med. 356 (10), 1020–1029. 10.1056/nejmoa067731 17296821

[B70] Mauri L. Kereiakes D. J. Yeh R. W. Driscoll-Shempp P. Cutlip D. E. Steg P. G. (2014). Twelve or 30 months of dual antiplatelet therapy after drug-eluting stents. N. Engl. J. Med. 371 (23), 2155–2166. 10.1056/nejmoa1409312 25399658 PMC4481318

[B142] McCarthy C. E. White J. M. Viola N. T. Gibson H. M. (2020). *In vivo* imaging technologies to monitor the immune system. Front.Immunol. 11, 1067 32582173 10.3389/fimmu.2020.01067 PMC7280489

[B71] McGoron A. Pratt R. Zhang J. Shiferaw Y. Thomas S. Millard R. (1994). Perfluorocarbon distribution to liver, lung and spleen of emulsions of perfluorotributylamine (FTBA) in pigs and rats and perfluorooctyl bromide (PFOB) in rats and dogs by 19F NMR spectroscopy. Artif. Cells, Blood Substitutes, Biotechnol. 22 (4), 1243–1250. 10.3109/10731199409138822 7849929

[B72] McInnes I. B. O'Dell J. R. (2010). State-of-the-art: Rheumatoid arthritis: Figure 1. Ann. rheumatic Dis. 69 (11), 1898–1906. 10.1136/ard.2010.134684 20959326

[B73] McInnes I. B. Schett G. (2007). Cytokines in the pathogenesis of rheumatoid arthritis. Nat. Rev. Immunol. 7 (6), 429–442. 10.1038/nri2094 17525752

[B74] Menz D-H. Feltgen N. Menz H. Müller B-K. Lechner T. Dresp J. (2018). How to ward off retinal toxicity of perfluorooctane and other perfluorocarbon liquids? Investigative Ophthalmol. Vis. Sci. 59 (12), 4841–4846. 10.1167/iovs.18-24698 30347078

[B75] Moasefi N. Fouladi M. Norooznezhad A. H. Yarani R. Rahmani A. Mansouri K. (2021). How could perfluorocarbon affect cytokine storm and angiogenesis in coronavirus disease 2019 (COVID-19): Role of hypoxia-inducible factor 1α. Inflamm. Res. 70 (7), 749–752. 10.1007/s00011-021-01469-8 34173853 PMC8233630

[B76] Mohanto N. Park Y-J. Jee J-P. (2023). Current perspectives of artificial oxygen carriers as red blood cell substitutes: A review of old to cutting-edge technologies using *in vitro* and *in vivo* assessments. J. Pharm. Investigation 53 (1), 153–190. 10.1007/s40005-022-00590-y PMC9344254 35935469

[B77] Morawski A. M. Winter P. M. Yu X. Fuhrhop R. W. Scott M. J. Hockett F. (2004). Quantitative “magnetic resonance immunohistochemistry” with ligand‐targeted 19F nanoparticles. Magnetic Reson. Med. An Official J. Int. Soc. Magnetic Reson. Med. 52 (6), 1255–1262. 10.1002/mrm.20287 15562481

[B78] Moriyasu F. Itoh K. (2009). Efficacy of perflubutane microbubble-enhanced ultrasound in the characterization and detection of focal liver lesions: Phase 3 multicenter clinical trial. Am. J. Roentgenol. 193 (1), 86–95. 10.2214/ajr.08.1618 19542399

[B79] Myerson J. He L. Lanza G. Tollefsen D. Wickline S. (2011). Thrombin‐inhibiting perfluorocarbon nanoparticles provide a novel strategy for the treatment and magnetic resonance imaging of acute thrombosis. J. Thrombosis Haemostasis 9 (7), 1292–1300. 10.1111/j.1538-7836.2011.04339.x PMC3686484 21605330

[B80] Myerson J. W. He L. Tollefsen D. M. Wickline S. A. (2010). Thrombin inhibitor perfluorocarbon nanoparticles for treatment and 19F tracking of acute thrombosis. J. Cardiovasc. Magnetic Reson. 12 (1), O60–O62. 10.1186/1532-429x-12-s1-o60

[B81] Numata K. Fukuda H. Morimoto M. Kondo M. Nozaki A. Oshima T. (2012). Use of fusion imaging combining contrast-enhanced ultrasonography with a perflubutane-based contrast agent and contrast-enhanced computed tomography for the evaluation of percutaneous radiofrequency ablation of hypervascular hepatocellular carcinoma. Eur. J. Radiology 81 (10), 2746–2753. 10.1016/j.ejrad.2011.11.052 22197088

[B82] Olaf M. Cooney R. (2017). Deep venous thrombosis. Emerg. Med. Clin. 35 (4), 743–770. 10.1016/j.emc.2017.06.003 28987427

[B83] Osório J. (2010). Long-term dual antiplatelet therapy and bleeding in stable patients—Insights from CHARISMA. Nat. Rev. Cardiol. 7 (9), 478. 10.1038/nrcardio.2010.114 20806464

[B84] Palmieri D. Brasili F. Capocefalo A. Bizien T. Angelini I. Oddo L. (2022). Improved hybrid-shelled perfluorocarbon microdroplets as ultrasound- and laser-activated phase-change platform. Colloids Surfaces A Physicochem. Eng. Aspects 641, 128522. 10.1016/j.colsurfa.2022.128522

[B85] Partlow K. C. Chen J. Brant J. A. Neubauer A. M. Meyerrose T. E. Creer M. H. (2007). 19F magnetic resonance imaging for stem/progenitor cell tracking with multiple unique perfluorocarbon nanobeacons. FASEB J. 21 (8), 1647–1654. 10.1096/fj.06-6505com 17284484

[B86] Paulissen S. M. van Hamburg J. P. Dankers W. Lubberts E. (2015). The role and modulation of CCR6+ Th17 cell populations in rheumatoid arthritis. Cytokine 74 (1), 43–53. 10.1016/j.cyto.2015.02.002 25828206

[B87] Pennati F. LoMauro A. D'Angelo M. G. Aliverti A. (2021). Non-invasive respiratory assessment in duchenne muscular dystrophy: From clinical research to outcome measures. Life (Basel) 11 (9), 947. 10.3390/life11090947 34575096 PMC8468718

[B88] Perricone C. Ceccarelli F. Matteo S. Di Carlo G. Bogdanos D. P. Lucchetti R. (2019). Porphyromonas gingivalis and rheumatoid arthritis. Curr. Opin. Rheumatology 31 (5), 517–524. 10.1097/bor.0000000000000638 31268867

[B89] Ravis W. R. Hoke J. F. Parsons D. L. (1991). Perfluorochemical erythrocyte substitutes: Disposition and effects on drug distribution and elimination. Drug metab. Rev. 23 (3-4), 375–411. 10.3109/03602539109029765 1935577

[B90] Robert F. (2010). The potential benefits of low-molecular-weight heparins in cancer patients. J. Hematol. Oncol. 3 (1), 3–12. 10.1186/1756-8722-3-3 20074349 PMC2830957

[B91] Roberts T. R. Harea G. T. Singha P. Sieck K. N. Beely B. M. Wendorff D. S. (2020). Heparin-free extracorporeal life support using tethered liquid perfluorocarbon: A feasibility and efficacy study. ASAIO J. 66 (7), 809–817. 10.1097/mat.0000000000001055 31453831

[B92] Ruiz‐Cabello J. Walczak P. Kedziorek D. A. Chacko V. P. Schmieder A. H. Wickline S. A. (2008). *In vivo* “hot spot” MR imaging of neural stem cells using fluorinated nanoparticles. Magnetic Reson. Med. An Official J. Int. Soc. Magnetic Reson. Med. 60 (6), 1506–1511. 10.1002/mrm.21783 PMC2597664 19025893

[B93] Sen Gupta A. (2017). Bio‐inspired nanomedicine strategies for artificial blood components. Wiley Interdiscip. Rev. Nanomedicine Nanobiotechnology. 9 (6), e1464. 10.1002/wnan.1464 PMC5599317 28296287

[B95] Siegel P. M. Chalupsky J. Olivier C. B. Bojti I. Pooth J-S. Trummer G. (2022). Early platelet dysfunction in patients receiving extracorporeal membrane oxygenation is associated with mortality. J. thrombosis thrombolysis 53 (3), 712–721. 10.1007/s11239-021-02562-9 PMC8444511 34529213

[B96] Song G. Ji C. Liang C. Song X. Yi X. Dong Z. (2017). TaOx decorated perfluorocarbon nanodroplets as oxygen reservoirs to overcome tumor hypoxia and enhance cancer radiotherapy. Biomaterials 112, 257–263. 10.1016/j.biomaterials.2016.10.020 27768978

[B97] Spahn D. (1999). Blood substitutes artificial oxygen carriers: Perfluorocarbon emulsions. Crit. Care 3 (5), R93–R97. 10.1186/cc364 11094488 PMC137239

[B98] Spuentrup E. Buecker A. Katoh M. Wiethoff A. J. Parsons E. C. Jr Botnar R. M. (2005). Molecular magnetic resonance imaging of coronary thrombosis and pulmonary emboli with a novel fibrin-targeted contrast agent. Circulation 111 (11), 1377–1382. 10.1161/01.cir.0000158478.29668.9b 15738354

[B99] Spuentrup E. Fausten B. Kinzel S. Wiethoff A. J. Botnar R. M. Graham P. B. (2005). Molecular magnetic resonance imaging of atrial clots in a swine model. Circulation 112 (3), 396–399. 10.1161/circulationaha.104.529941 16009790

[B100] Stanton S. E. Eary J. F. Marzbani E. A. Mankoff D. Salazar L. G. Higgins D. (2016). Concurrent SPECT/PET-CT imaging as a method for tracking adoptively transferred T-cells *in vivo* . J. Immunother. cancer 4 (1), 1–5. 10.1186/s40425-016-0131-3 PMC4869363 27190628

[B101] Stein P. D. Beemath A. Olson R. E. (2005). Obesity as a risk factor in venous thromboembolism. Am. J. Med. 118 (9), 978–980. 10.1016/j.amjmed.2005.03.012 16164883

[B102] Stoll G. Basse‐Lüsebrink T. Weise G. Jakob P. (2012). Visualization of inflammation using 19F‐magnetic resonance imaging and perfluorocarbons. Wiley Interdiscip. Rev. Nanomedicine Nanobiotechnology 4 (4), 438–447. 10.1002/wnan.1168 22422659

[B103] Sun Y. Zhao D. Wang G. Wang Y. Cao L. Sun J. (2020). Recent progress of hypoxia-modulated multifunctional nanomedicines to enhance photodynamic therapy: Opportunities, challenges, and future development. Acta Pharm. Sin. B 10 (8), 1382–1396. 10.1016/j.apsb.2020.01.004 32963938 PMC7488364

[B104] Svanström H. Lund M. Melbye M. Pasternak B. (2018). Concomitant use of low-dose methotrexate and NSAIDs and the risk of serious adverse events among patients with rheumatoid arthritis. Pharmacoepidemiol Drug Saf. 27 (8), 885–893. 10.1002/pds.4555 29797447

[B105] Swider E. Daoudi K. Staal A. H. Koshkina O. Van Riessen N. K. van Dinther E. (2018). Clinically-applicable perfluorocarbon-loaded nanoparticles for *in vivo* photoacoustic, 19F magnetic resonance and fluorescent imaging. Nanotheranostics 2 (3), 258–268. 10.7150/ntno.26208 29868350 PMC5984288

[B106] Szíjjártó C. Rossi S. Waton G. Krafft M. P. (2012). Effects of perfluorocarbon gases on the size and stability characteristics of phospholipid-coated microbubbles: Osmotic effect versus interfacial film stabilization. Langmuir 28 (2), 1182–1189. 10.1021/la2043944 22176688

[B107] Szilagyi J. T. Avula V. Fry R. C. (2020). Perfluoroalkyl substances (PFAS) and their effects on the placenta, pregnancy, and child development: A potential mechanistic role for placental peroxisome proliferator–activated receptors (PPARs). Curr. Environ. Health Rep. 7 (3), 222–230. 10.1007/s40572-020-00279-0 32812200 PMC7473499

[B108] Taghizadeh B. Ghavami L. Derakhshankhah H. Zangene E. Razmi M. Jaymand M. (2020). Biomaterials in valvular heart diseases. Front. Bioeng. Biotechnol. 8, 529244. 10.3389/fbioe.2020.529244 33425862 PMC7793990

[B109] Tak S. Barraclough M. (2018). ‘Pseudo-calcifications’: Detection of perfluorocarbon residue on a computed tomography scan 15 years after liquid ventilation therapy at 3 months of age. BMJ Case Rep. 2018, bcr2017223958–2017-223958. 10.1136/bcr-2017-223958 PMC5848008 29507033

[B110] Takahashi M. Hasegawa K. Arita J. Hata S. Aoki T. Sakamoto Y. (2012). Contrast-enhanced intraoperative ultrasonography using perfluorobutane microbubbles for the enumeration of colorectal liver metastases. J. Br. Surg. 99 (9), 1271–1277. 10.1002/bjs.8844 22829436

[B111] Tang Q. Cui J. Tian Z. Sun J. Wang Z. Chang S. (2017). Oxygen and indocyanine green loaded phase-transition nanoparticle-mediated photo-sonodynamic cytotoxic effects on rheumatoid arthritis fibroblast-like synoviocytes. Int. J. Nanomedicine 12, 381–393. 10.2147/ijn.s120902 28123298 PMC5234560

[B112] Tarighatnia A. Fouladi M. R. Nader N. D. Aghanejad A. Ghadiri H. (2022). Recent trends of contrast agents in ultrasound imaging: A review of the classifications and applications. Mater. Adv. 3 (9), 3726–3741. 10.1039/d1ma00969a

[B113] Temme S. Grapentin C. Quast C. Jacoby C. Grandoch M. Ding Z. (2015). Noninvasive imaging of early venous thrombosis by 19F magnetic resonance imaging with targeted perfluorocarbon nanoemulsions. Circulation 131 (16), 1405–1414. 10.1161/circulationaha.114.010962 25700177

[B114] Tennstaedt A. Mastropietro A. Nelles M. Beyrau A. Hoehn M. (2015). *In vivo* fate imaging of intracerebral stem cell grafts in mouse brain. PLoS One 10 (12), e0144262. 10.1371/journal.pone.0144262 26641453 PMC4671578

[B115] Toyama H. Takada M. Tanaka T. Suzuki Y. Kuroda Y. (2003). Characterization of islet-infiltrating immunocytes after pancreas preservation by two-layer (UW/perfluorochemical) cold storage method. Transplant. Proc. 35 (4), 1503–1505. 10.1016/s0041-1345(03)00370-1 12826205

[B116] Tran T. D. Caruthers S. D. Hughes M. Marsh J. N. Cyrus T. Winter P. M. (2007). Clinical applications of perfluorocarbon nanoparticles for molecular imaging and targeted therapeutics. Int. J. nanomedicine 2 (4), 515–526. 18203420 PMC2676820

[B117] Uemura H. Sano F. Nomiya A. Yamamoto T. Nakamura M. Miyoshi Y. (2013). Usefulness of perflubutane microbubble-enhanced ultrasound in imaging and detection of prostate cancer: Phase II multicenter clinical trial. World J. urology 31, 1123–1128. 10.1007/s00345-012-0833-1 22311543

[B118] Viatte S. Plant D. Raychaudhuri S. (2013). Genetics and epigenetics of rheumatoid arthritis. Nat. Rev. Rheumatol. 9 (3), 141–153. 10.1038/nrrheum.2012.237 23381558 PMC3694322

[B119] Vidallon M. L. P. Giles L. W. Pottage M. J. Butler C. S. G. Crawford S. A. Bishop A. I. (2022). Tracking the heat-triggered phase change of polydopamine-shelled, perfluorocarbon emulsion droplets into microbubbles using neutron scattering. J. Colloid Interface Sci. 607, 836–847. 10.1016/j.jcis.2021.08.162 34536938

[B120] Virani S. S. Alonso A. Benjamin E. J. Bittencourt M. S. Callaway C. W. Carson A. P. (2020). Heart disease and stroke statistics-2020 update: A report from the American heart association. Circulation 141 (9), e139–e596. 10.1161/CIR.0000000000000757 31992061

[B121] Vu-Quang H. Vinding M. S. Jakobsen M. Song P. Dagnaes-Hansen F. Nielsen N. C. (2019). Imaging rheumatoid arthritis in mice using combined near infrared and (19)F magnetic resonance modalities. Sci. Rep. 9 (1), 14314. 10.1038/s41598-019-50043-0 31586092 PMC6778085

[B122] Wang H. Li J. Wang Y. Gong X. Xu X. Wang J. (2020). Nanoparticles-mediated reoxygenation strategy relieves tumor hypoxia for enhanced cancer therapy. J. Control. Release 319, 25–45. 10.1016/j.jconrel.2019.12.028 31862359

[B123] Wikström S. Lindh C. H. Shu H. Bornehag C-G. (2019). Early pregnancy serum levels of perfluoroalkyl substances and risk of preeclampsia in Swedish women. Sci. Rep. 9 (1), 9179. 10.1038/s41598-019-45483-7 31235847 PMC6591359

[B124] Wrobeln A. Laudien J. Groß-Heitfeld C. Linders J. Mayer C. Wilde B. (2017). Albumin-derived perfluorocarbon-based artificial oxygen carriers: A physico-chemical characterization and first *in vivo* evaluation of biocompatibility. Eur. J. Pharm. Biopharm. 115, 52–64. 10.1016/j.ejpb.2017.02.015 28232105

[B125] Wu L. Liu F. Liu S. Xu X. Liu Z. Sun X. (2020). <p&gt;Perfluorocarbons-Based <sup&gt;19&lt;/sup&gt;F Magnetic Resonance Imaging in Biomedicine&lt;/p&gt;. Int. J. Nanomedicine 15, 7377–7395. 10.2147/ijn.s255084 33061385 PMC7537992

[B126] Wu L. Wen X. Wang X. Wang C. Sun X. Wang K. (2018). Local intratracheal delivery of perfluorocarbon nanoparticles to lung cancer demonstrated with magnetic resonance multimodal imaging. Theranostics 8 (2), 563–574. 10.7150/thno.21466 29290827 PMC5743567

[B127] Wu Y. Vazquez-Prada K. X. Liu Y. Whittaker A. K. Zhang R. Ta H. T. (2021). Recent advances in the development of theranostic nanoparticles for cardiovascular diseases. Nanotheranostics 5 (4), 499–514. 10.7150/ntno.62730 34367883 PMC8342263

[B128] Xavierselvan M. Cook J. Duong J. Diaz N. Homan K. Mallidi S. (2022). Photoacoustic nanodroplets for oxygen enhanced photodynamic therapy of cancer. Photoacoustics 25, 100306. 10.1016/j.pacs.2021.100306 34917471 PMC8666552

[B129] Xiang Y. Bernards N. Hoang B. Zheng J. Matsuura N. (2019). Perfluorocarbon nanodroplets can reoxygenate hypoxic tumors *in vivo* without carbogen breathing. Nanotheranostics 3 (2), 135–144. 10.7150/ntno.29908 31008022 PMC6470341

[B130] Yang J. Li Y. Sun J. Zou H. Sun Y. Luo J. (2022). An osimertinib-perfluorocarbon nanoemulsion with excellent targeted therapeutic efficacy in non-small cell lung cancer: Achieving intratracheal and intravenous administration. ACS Nano 16 (8), 12590–12605. 10.1021/acsnano.2c04159 35863049

[B131] Yang Z. Tao D. Zhong W. Liu Z. Feng L. Chen M. (2022). Perfluorocarbon loaded fluorinated covalent organic polymers with effective sonosensitization and tumor hypoxia relief enable synergistic sonodynamic-immunotherapy. Biomaterials 280, 121250. 10.1016/j.biomaterials.2021.121250 34823883

[B133] Ye J. Wang Q. Zhou X. Zhang N. (2008). Injectable actarit-loaded solid lipid nanoparticles as passive targeting therapeutic agents for rheumatoid arthritis. Int. J. Pharm. 352 (1-2), 273–279. 10.1016/j.ijpharm.2007.10.014 18054182

[B134] Zhang H. Chen J. Zhu X. Ren Y. Cao F. Zhu L. (2018). Ultrasound induced phase-transition and invisible nanobomb for imaging-guided tumor sonodynamic therapy. J. Mater Chem. B 6 (38), 6108–6121. 10.1039/c8tb01788c 32254821

[B135] Zhou H. F. Chan H. W. Wickline S. A. Lanza G. M. Pham C. T. (2009). α _v_ β _3_ –Targeted nanotherapy suppresses inflammatory arthritis in mice. Faseb J. 23 (9), 2978–2985. 10.1096/fj.09-129874 19376816 PMC2735365

[B136] Zhou H. F. Hu G. Wickline S. A. Lanza G. M. Pham C. T. (2010). Synergistic effect of antiangiogenic nanotherapy combined with methotrexate in the treatment of experimental inflammatory arthritis. Nanomedicine (Lond). 5 (7), 1065–1074. 10.2217/nnm.10.78 20874021 PMC3035945

[B137] Zhou H. F. Yan H. Senpan A. Wickline S. A. Pan D. Lanza G. M. (2012). Suppression of inflammation in a mouse model of rheumatoid arthritis using targeted lipase-labile fumagillin prodrug nanoparticles. Biomaterials 33 (33), 8632–8640. 10.1016/j.biomaterials.2012.08.005 22922023 PMC3583210

[B138] Zhou J. Xue C. Hou Y. Li M. Hu Y. Chen Q. (2019). Oxygenated theranostic nanoplatforms with intracellular agglomeration behavior for improving the treatment efficacy of hypoxic tumors. Biomaterials 197, 129–145. 10.1016/j.biomaterials.2019.01.002 30641264

[B139] Zhou Z. Zhang B. Zai W. Kang L. Yuan A. Hu Y. (2019). Perfluorocarbon nanoparticle-mediated platelet inhibition promotes intratumoral infiltration of T cells and boosts immunotherapy. Proc. Natl. Acad. Sci. 116 (24), 11972–11977. 10.1073/pnas.1901987116 31142648 PMC6575596

[B140] Zhou Z. Zhang B. Zhang H. Huang X. Hu Y. Sun L. (2009). Drug packaging and delivery using perfluorocarbon nanoparticles for targeted inhibition of vascular smooth muscle cells. Acta Pharmacol. Sin. 30 (11), 1577–1584. 10.1038/aps.2009.146 19890365 PMC4003007

[B141] Zhu B. Wang L. Huang J. Xiang X. Tang Y. Cheng C. (2019). Ultrasound-triggered perfluorocarbon-derived nanobombs for targeted therapies of rheumatoid arthritis. J. Mater. Chem. B 7 (29), 4581–4591. 10.1039/c9tb00978g

